# Should I vote-by-mail or in person? The impact of COVID-19 risk factors and partisanship on vote mode decisions in the 2020 presidential election

**DOI:** 10.1371/journal.pone.0274357

**Published:** 2022-09-15

**Authors:** Lonna Rae Atkeson, Wendy L. Hansen, Maggie Toulouse Oliver, Cherie D. Maestas, Eric C. Wiemer

**Affiliations:** 1 Department of Political Science, Florida State University, Tallahassee, Florida, United States of America; 2 Department of Political Science, University of New Mexico, Albuquerque, New Mexico, United States of America; 3 New Mexico Secretary of State, Santa Fe, New Mexico, United States of America; 4 Department of Political Science, Purdue University, West Lafayette, Indiana, United States of America; Vanderbilt University, UNITED STATES

## Abstract

While the evidence is clear that 2020 voters shifted away from Election Day voting in favor of vote-by-mail and early voting, we know very little about how health risk versus party polarization around risk assessment influenced how and when to vote. We rely on individual-level observational data in the form of high-quality official voter administrative records from the State of New Mexico to ask how pandemic-related risk factors, especially voter age along with partisanship influenced voter decision-making. To identify causal factors, we use a difference-in-differences design and hazard model that compare 2020 general election and primary voter behavior to 2018 and 2016. We find that age and party were large factors in vote mode decisions in 2020, but not in 2016 or 2018. We consider the implications of our findings on how health risk and partisanship interact to influence decision-making.

## Introduction

In most elections, voters make routine choices about their method of voting based on relatively straightforward assessments of the costs and benefits, including the value of convenience, that comes from voting in-person early, in-person on Election Day or by mail [1–3]. However, the choice of when and how to vote in the 2020 presidential election was anything but routine, as a pandemic swept across the nation during a hotly contested presidential election. COVID-19 introduced a new potential cost of voting in person–exposing oneself or possibly loved ones to the risk of illness and death.

States took very seriously the need to provide a healthy election environment, and many altered typical election practices to de-densify polling places and to make mail balloting easier, [4]. The Center for Disease Control (CDC) encouraged voters to use, “voting alternatives that limit the number of people you come in contact with or the amount of time you are in contact with others” [5].

The pandemic led Congressional Democrats to introduce legislation to expand no excuse VBM and early voting in all the states [6], but messages from GOP elites, especially President Trump, highlighted concerns that ballots cast remotely by mail could result in lost, fraudulent, or miscounted votes [7, 8]. The advantage of voting in person is that most voters get to place their ballot into the voting machine and hence observe their ballot being accepted and counted. Vote- by-mail (VBM) naturally introduces uncertainty since the voter must rely on unobserved third parties to ensure their ballot arrives and is counted [9]. In this case, partisan-based elite messaging and polarized media content magnified the uncertainty about whether the benefits attached to voting would be realized using VBM, and this message resonated more strongly with Republicans than Democrats [10, 11].

These competing risks increased the complexity of voters’ choices among vote modes as they had to weigh the reduced risk of COVID-19 infection against the risk of something going wrong with their mail ballot. While evidence is clear that 2020 voters shifted away from Election Day voting in favor of VBM and early voting [12], we know very little about how health risk versus partisanship influenced risk assessment on vote mode choice, a question we ask and answer here.

Unlike other studies that have relied on survey data or aggregate observational data to examine differences in preferences for COVID-19 related policies and behaviors [13, 14], including mail balloting [11, 15, 16], we rely on individual-level observational data in the form of high-quality official voter administrative records from the State of New Mexico (SNM) to ask how pandemic-related risk factors, especially voter age and partisanship, influenced whether voters decided to VBM or to vote in person and when they decided to do so [but see 17].

Administrative data are important to analyze because self-reported behavior is fungible and less reliable; social desirability plays a role in people’s survey responses allowing them to appear more behaviorally consistent with their world view than they are [18, 19]. For example, in a recent study by Smith et al. [20], Trump voters in Florida were more likely to misremember their previous VBM experiences and more likely to misestimate how they planned on voting in the 2020 election than Biden voters. Attitudes and behavior, while related, are not perfectly correlated. Therefore, analysis of documented behavior may prove more reliable and valid for revealing patterns of choice and may differ from conclusions drawn solely from self-expressed behaviors from surveys. At the very least, high quality administrative data offers an alternative and potentially corroborative test to important social questions of the day.

To clarify differences in decision-making in 2020, we use panel data and a difference-in-differences (DID) model approach that allows us to compare 2020 voting mode behavior with 2018 and 2016 for the same individuals across the parties and counties who faced different levels of post-pandemic risk as measured by age. We also employ a hazard model to identify the average number of days that in-person voters waited after early voting opened in 2018 and 2020. The administrative data has the necessary variables and the universe of participants to examine age-based risk and partisanship. Importantly the high-quality nature of the data provides strong external validity, while the modeling approaches allow for greater causal inference and add greater internal validity to our research design. Importantly, during the three election cycles the same voting machines were used across the state, and the number and location of early and Election Day vote centers were nearly identical. Thus, the major differences in voters’ selection of vote mode were the pandemic and the impact of elite messaging regarding the best way to vote.

We expect observable patterns in the 2020 vote-mode data to be consistent with a process of partisan-based selective uptake of risk messages prevalent during the voting period. Risk assessments affect both the “costs” associated with casting a ballot using a particular mode and the “benefits” from casting a ballot, including the satisfaction derived from having one’s vote counted. While we cannot directly observe individual-level uptake of risk information in the administrative data, the assumptions that ground our expectations are derived from well-tested theories of how individuals seek and process information in extraordinary moments in society [21–23].

Catastrophic events, such as the COVID-19 pandemic, create shock and anxiety that stimulate people to seek and process information about risks with greater accuracy as they assess their day-to-day choices in light of potential harms [21]. In such situations, individuals search for correct answers rather than answers that support a predisposed position because accuracy is critical to averting imminent danger [24]. Assessments of risk in the earliest days of catastrophes tends to be the least partisan because journalists and the public turn to common sources of expert information [25]. However, over time, political elites seek to shift blame, often in partisan ways, and to frame events in ways that are politically beneficial [26]. When elites are polarized and media is fragmented along party lines, framing and risk messages diverge. Analysis of news media during the pandemic shows early polarization in media and elite messaging that was measurable by March 2020 and persisted through the election [27].

Given the broad availability of information about the virus in multiple news and non-news sources, it is reasonable to assume voters were aware that COVID-19 risk levels varied by age. Two of the three primary factors for COVID-19 complications, including death, are age (since older adults are more at risk than younger adults) and other medical conditions, which often correlate with age [28]. The third risk factor is pregnancy, which we cannot control for.

**Fig 1:**
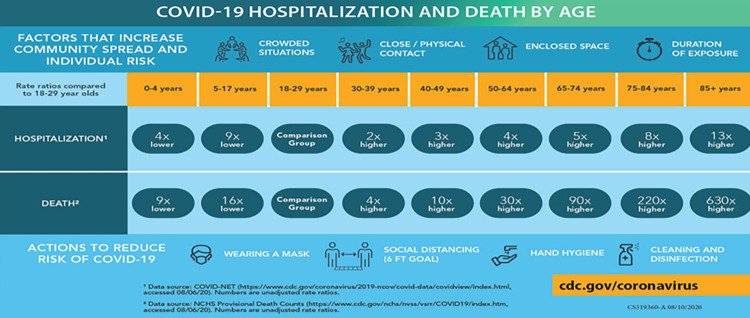
Centers for disease control graph describing COVID-19 hospitalization and death by age [29].

Fig 1 shows one example of a CDC graphic dated 08/10/2020 that arose from the Google search string “risk and covid” during the election. It displays the risk of hospitalization and death from COVID-19 by age. For example, compared to the youngest group of adult cohorts (ages 18–29), people age 85+ are 13 times more likely to be hospitalized and 630 times more likely to die. We mirror these age categories in our analyses to test the relationship between COVID-19 risk and behavior. Notably, Fig 1 also shows situations that are likely to increase individual risk, focusing on “crowded situations, close physical contact, and enclosed space.” Each of these factors is associated with in-person voting and would under typical conditions create greater density especially during Election Day voting and the last few days of early voting when early turnout historically soars.

With such information permeating society, *we expect that more voters should choose to VBM in 2020 than in previous elections* **(hypothesis 1)**, *and the effect of age is stronger in 2020 than in previous elections*. *Thus*, *we expect older voters should be more likely to make that choice than younger voters* **(hypothesis 2a)**.

At the same time, the fragmented and polarized media environment gave rise to variation in health and ballot risk perceptions across similarly aged individuals. Although people may seek “accurate” information in catastrophes, they often seek information from trusted sources, such as co-partisans, rather than from the full range of possible sources [30]. Content analysis of network and newspaper stories during the spring of 2020 revealed highly polarized and politicized messaging around COVID-19, where the framing and emphasis varied across party and news sources [31, 32].

There is also evidence that individuals engaged in partisan motivated reasoning. For example, Druckman et al. [13] show a strong correlation between out-party animosity and citizen attitudes toward the pandemic and pandemic related policies. A national survey in March 2020 showed substantial differences in risk perceptions by Republicans and Democrats, and that those who consumed right-leaning media perceived health risks from COVID-19 to be lower, independent of partisanship [33]. Nationwide surveys conducted in June 2020 by Pew Research Center showed substantial polarization between Democrats and Republicans regarding their attitudes towards mask wearing, risk of infection, and comfort with activities such as going to the grocery store, eating out, attending sporting events, or a going to a hair salon [34].

Perceptions of risks associated with mail balloting were also highly polarized by party. President Trump, for example, took a strong position against “universal mail balloting” in which states automatically send ballots out to each eligible voter, and he began tweeting about potential problems in mail balloting as early as April 8 [19]. Thus, a practice that was largely nonpartisan before the 2020 election became rapidly polarized along party lines in the months leading up to it [11, 16, 35].

Therefore, while health risk factors such as age should be related to voters’ choice of especially VBM, we expect to also observe vote patterns consistent with partisan-based motivated reasoning that serve to encourage in-person voting for those concerned about VBM risks, and note that more spaced out early voting offers the safest of the in-person voting options because it reduces polling place density that directly allows for greater social distancing during the pandemic. Early voting was encouraged by the CDC to mitigate health risks associated with crowded election-day vote centers [36]. Taken together, we expect a partisan-based risk mitigation strategy to lead to the following observable outcomes during the pandemic in 2020 compared to prior election years:

*Democrats and independents should*, *on average*, *be more likely to VBM than Republicans* in 2020 compared to prior election years **(hypothesis 3a)**. *Democrats should*, *on average*, *be more likely to VBM than Independents* (**hypothesis 3b**) given their more homogeneous messaging environment. And, given Republicans’ greater concern about the risk of mail-balloting to fraud in 2020, we *expect Republicans*, *on average*, *to be more likely to vote in-person early than Democrats and Independents* **(hypothesis 3c)**. Finally, we expect that the partisan-based messaging that balanced COVID-19 health-risks against VBM risks to moderate the effect of age and year on vote-mode choice such that the effect of age on vote mode choice is less for Republicans than Independents, and Democrats (**hypothesis 3d**).

Because the CDC encouraged more spaced-out voting during early voting, our argument also has temporal implications for the 2020 in-person vote: *we expect voters who choose to vote in person to*, *on average*, *vote earlier in the process compared to the prior election* (**hypothesis 4a**). Given older voters’ greater likelihood of COVID-19 health risks, we also expect that they will be more responsive to the early voting messaging in 2020 and therefore *older voters who vote early will be more likely to vote earlier than younger voters* (**hypothesis 4b**).

## Data and methods

Our data source is the administrative records of voting from the New Mexico Secretary of State’s (NMSOS) office, which consists of all voters listed in the voter registration file for each election. A review by the Florida State University Office for Human Subject Protection determined that, because we rely on administrative data for our research design, it does not meet the definition of human subjects’ data as defined by DHHS and/or FDA regulations; thus, IRB review and approval for its use is not required.

To test hypotheses 1-3d about vote-mode choice, we estimate the change in voting mode behavior with a panel difference-in-differences analysis using logistic regression models of voters’ individual vote-mode decisions in New Mexico (NM) in 2020 compared to those made in 2016 and 2018. This resulted in a general election model size of 312,472 in each general election for a total number of observations of 937,416 across the 3 elections and 89,816 voters in each primary for a total of 269,448 across the 3 elections. The DID model is a quasi-experimental approach to estimating the effects of the pandemic on vote mode choice at different levels of our main risk proxies–age and partisanship–in the pre- and post-pandemic contexts.

In the *Supplemental Information* section (S1 and S2 Figs), we also show the graphs for the general election cross-sectional DID model that uses the full electorate for each election (2020 General Election N = 922,840, Primary N = 402,785; 2018 General Election N = 698,202 Primary N = 309,340, and 2016 General Election N = 801,168, Primary N = 246,054). These results corroborate our panel findings and show that what we see is a product of the differences caused by the pandemic and the polarization around it.

We test hypotheses 4a and 4b using a Hazard model that includes all in-person voters, which allows us to model their vote-date (N = 380,930).

Voter registration data are high quality, meaning they are both reliable and valid, and below we describe how the data are collected and maintained. Importantly, these data provide an accurate or valid account of how each voter chose to cast their ballot and gets us away from socially desirable and potentially inaccurate survey -responses. From the voter file, we have individual data on each voter’s vote mode along with their county, age, sex, and party affiliation. In the 2020 and 2018 general elections we also know what day in-person voters went to the polls. These data provide almost all the necessary variables to test our hypotheses and, given the reliable and valid nature of the individual voter data, provide strong external validity, while the comparative nature of our tests and use of a panel difference-in-differences analysis, across election years, provides strong internal validity allowing us greater purchase on causal inference.

### Case selection

NM is an excellent case to examine the relationship between age, partisanship and vote mode decisions because NM does not limit voting options and allows voters to easily cast a ballot by mail or in person. Voter choice is a critical administrative process necessary for understanding how risk and party polarization influences vote mode decision-making. NM has “no excuse” absentee voting laws, which makes VBM ballots easily available to every eligible elector. In NM, voters must fill out an absentee ballot application to receive a ballot and this request must be made each election. Thus, the default position is that voters will vote in person, unless they request a mail ballot. In 2020, large VBM campaigns across the state by both Democratic and Republican groups and by election administrators resulted in VBM applications being mailed to all qualified electors, lowering the cost of obtaining a VBM ballot. Access to VBM was easy—voters could fill out an online application to VBM, mail in the application, email it, or make the request by phone or in person at their county clerk’s office, and VBM ballots were postage prepaid, further decreasing the costs associated with VBM.

NM also makes it easy for voters to vote in person before Election Day. NM has a robust early in-person voting system that begins 28 days before the general election in the County Clerk’s Office. At 21 days before the election this process expands to a larger number of locations across each county, thus reducing travel costs associated with voting early at an in-person voting location. On Election Day this process expands even further with even more vote centers opening to accommodate Election Day turnout. Importantly, this process was not substantially affected by the pandemic. Thus, unlike many states that changed their election system specifically for COVID-19, NM provides a state environment in which the voting mode choices were substantively the same in 2020 as they were in 2016 and 2018 allowing us to observe and compare how the 2020 context shaped and changed voter decision-making.

### Overview of voter data maintenance and security in New Mexico

The data for this study were drawn from data compiled by the NMSOS in accordance with the laws and statues of the SNM. These laws specify when and how data on voters are collected, updated, and validated, and ensure that the process that generated the data in each election period used in our study is the same. The SNM utilizes a centralized, state-wide voter registration and election management system, termed the Statewide Election Registration and Voter Information System (SERVIS), which is administered by the NMSOS. The NMSOS is responsible for overseeing and maintaining the system, and, in some cases providing voter registration list maintenance data to the state’s 33 county clerks, who administer elections at the county jurisdictional level, for processing. For details on NM laws that govern the collection and maintenance of voter data, see Help America Vote Act (HAVA) of 2002 (52 USC Ch. 209 §21083), New Mexico State Statute Chapter 1, NMSA 1978 and see https://www.sos.state.nm.us/voting-and-elections/how-we-secure-your-vote/.

County Clerks in NM are responsible for actual voter list maintenance activities, including adding, removing, and updating voter registration records; posting voting credit; entering data regarding the status of and applications for VBM ballots and the disposition of those ballots; flagging of returned mail; and other related entries, and are required by law to regularly update that information in SERVIS, based on statutory and procedural requirements.

Per information received directly from the NMSOS, SERVIS is maintained on a secure, cloud-based server by the NMSOS and is protected by election security best practices to protect data from being hacked or tampered with. NM requires multi-factor authentication to access the state-wide system, and only certified election officials at the state or county level, or certified vendors, are provided with access to the system.

At the county level, all voter and election-related data are ultimately maintained within SERVIS, however, every county utilizes a pre-approved “ballot-on-demand” system provided by a state-certified vendor, and connected in “real time” (i.e. within a 2 minute period, depending upon connectivity speed) to SERVIS, in order to engage in election-time related tasks such as: processing (accepting/rejecting) applications for VBM ballots; tracking mailing, receipt, and acceptance (i.e. counting) of those ballots; issuance of early voting and Election Day ballots; and tracking the issuance/cancellation of spoiled, replacement, or provisional ballots in any vote mode, to voters. Once the data are collected in the “ballot-on-demand” system, they are uploaded in real-time to SERVIS. This process of data collection and maintenance in the SNM ensures the availability of the high quality and consistent data that are used in this study.

### Model considerations

We test our hypotheses using panel data from voters who participated in all three federal elections, 2016, 2018, and 2020, matched across party, age category, and county. In the hazard model we also match on in-person voting. Matching reduces the chance that unmeasured confounders that correlate with party, age, and location affect outcomes across elections, and ensures that the differences we see in 2020 are the result of the pandemic context and not the result of differences in the groups of voters participating in the election. The underlying assumption is that the vote mode choices for these individuals would not change much from election to election in the absence of the pandemic. Therefore, we expect individual characteristics like age and party to have little systematic influence on vote mode choice prior to the 2020 elections, but these variables take on special meaning during 2020 because of the pandemic context when they become proxies for individual exposure to risk. As a result, we expect greater average marginal effects of age and party on vote mode choice in 2020 compared to prior election years.

The pandemic context is the basis of the “treatment effect,” but because the pandemic affected everyone, we do not have a true binary “control” group in 2020 for comparison. Rather, we have different levels of health risk during the pandemic, measured by age and moderated by party considerations. Thus, one key quantity of interest in 2020 is simply the average causal response across age-party categories in 2020. The second quantity of interest is the difference in those effects on vote mode selection across years. In other words, we explore whether the effects of age-party categories differ from one election to the next.

To be consistent with elite messaging on age, we measure age using categories as defined by the CDC in Fig 1. To examine the role of partisanship, we rely on voters’ stated party identifications in the administrative voter files, defined as Democrat, Decline-to-State/No-Major Party/Independent, and Republican. Party identification is a meaningful category in NM because NM is a closed primary state during these three elections and therefore voters must be registered partisans to participate in primary elections. We interact party with age categories to identify how partisanship and age risk factors worked together to explain behavior in the pre- and post-pandemic contexts.

Empirically, the model we estimate is a logit model where our dependent variable is a binary vote mode choice (e.g., VBM versus alternatives), and our treatment variable is election year (2016, 2018, 2020) multiplied by party (Democrat, Independent, or Republican) and CDC age categories. Standard errors are clustered by the voter. Decomposing the results of the interaction allows us to see whether the differences in the conditional effects of age by party differ between pre pandemic and pandemic election pairs (2020 vs 2016 and 2020 vs 2018) compared to an election pair with no pandemic to activate the “treatments” of age and party (2018 to 2016). We also formally test whether the change in effect of the election year context differs by party within each age category.

We include standard controls for gender and for imputed race and ethnicity using Imai and Khanna’s [37] method of considering both last name and location of the voter. We include fixed effects for county since election administration implementation happens at this level and cluster by the individual voter. We show multiple types of graphs to show both the change in the predicted probability of selecting each vote mode by age and party as well as the difference-in-differences between party groups across years in each age category. We also run a multinomial logit on voting mode and show age and party effects by election year to observe the predicted probabilities and understand the substantive size of the effects in each election context. All models were estimated in Stata MP 17.

In the Supplemental Information section, S1–S3 Tables present descriptive information about our variables for each model. S4–S16 Tables present the model coefficients to these graphs and S1 and S2 Figs present the difference in the marginal effects for the cross-sectional DID model.

## Results

### Vote mode trend

**Fig 2:**
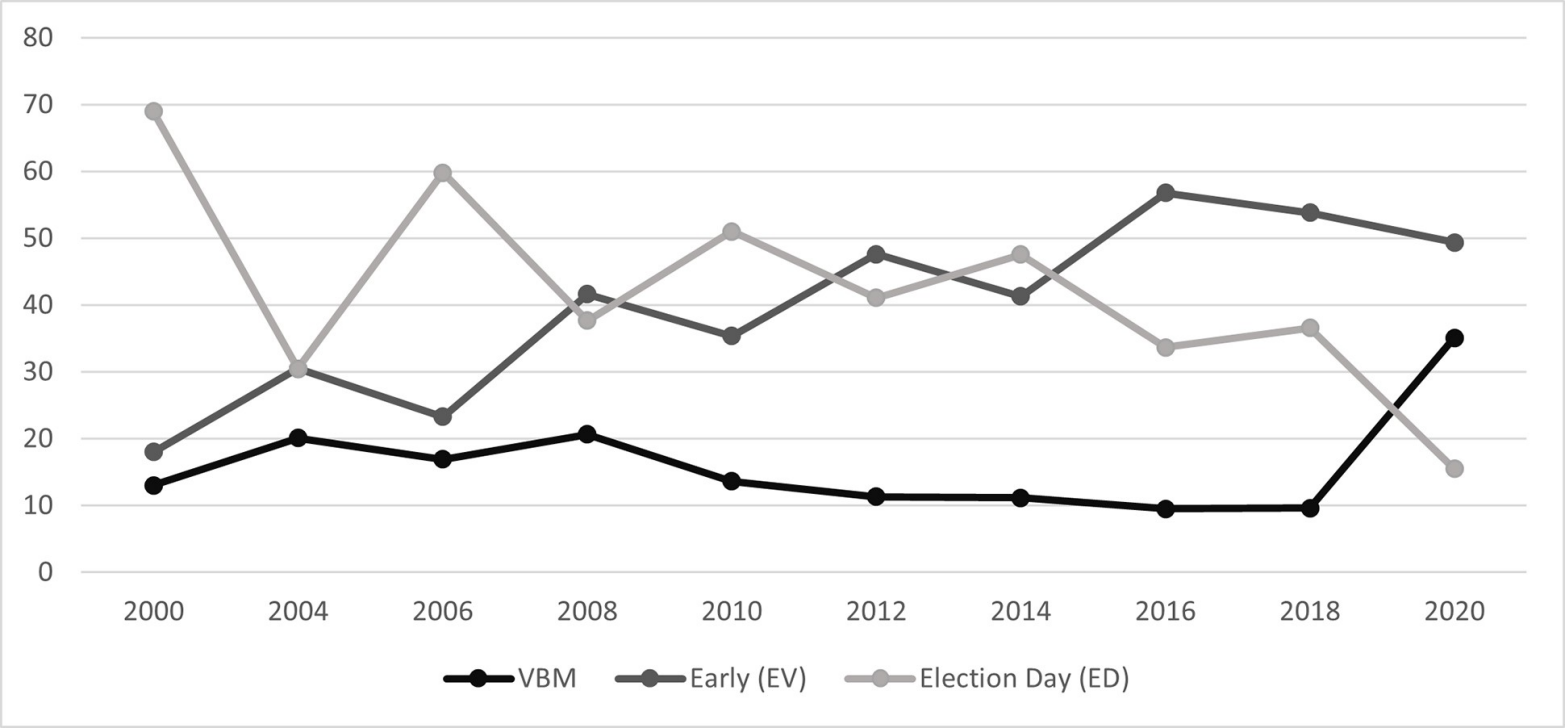
NM voting mode over time.

Fig 2 presents election trend data that supports the first hypothesis that the pandemic encouraged more mail and early in-person voting than Election Day voting compared to prior election cycles. Historically, VBM has not been very popular in NM, averaging only about 10% between 2010 and 2018. Thus, changes we see in 2020 are primarily due to changes caused by the pandemic. While in-person voting in 2020 remained the mode, comparing 2020 to 2018 and 2016 respectively, Election Day (16% vs 37% vs 34%) and early voting (49% vs 54% vs 57%) declined substantially, while VBM significantly increased (35% vs 10% vs 10%).

### General election vote mode

The administrative data allow us to estimate subgroup treatment effects for different group-based “dosages” of risk in a pandemic versus pre-pandemic time-period. This allows us to see how risk context, as conveyed largely through elite messaging about health and politics, affected the probability of selecting one vote mode over another. The empirical design assumes parallel trends such that voter behavior in the treated category, if untreated, would have followed a similar trend as prior years. Though not directly testable, we find that the 2016 and 2018 vote mode choice is very similar across these groups which provides evidence that consistency across elections is a plausible assumption. However, once party polarization around mail balloting and health risks from COVID-19 emerge in 2020, we see changes in behavior consistent with our hypotheses.

**Fig 3:**
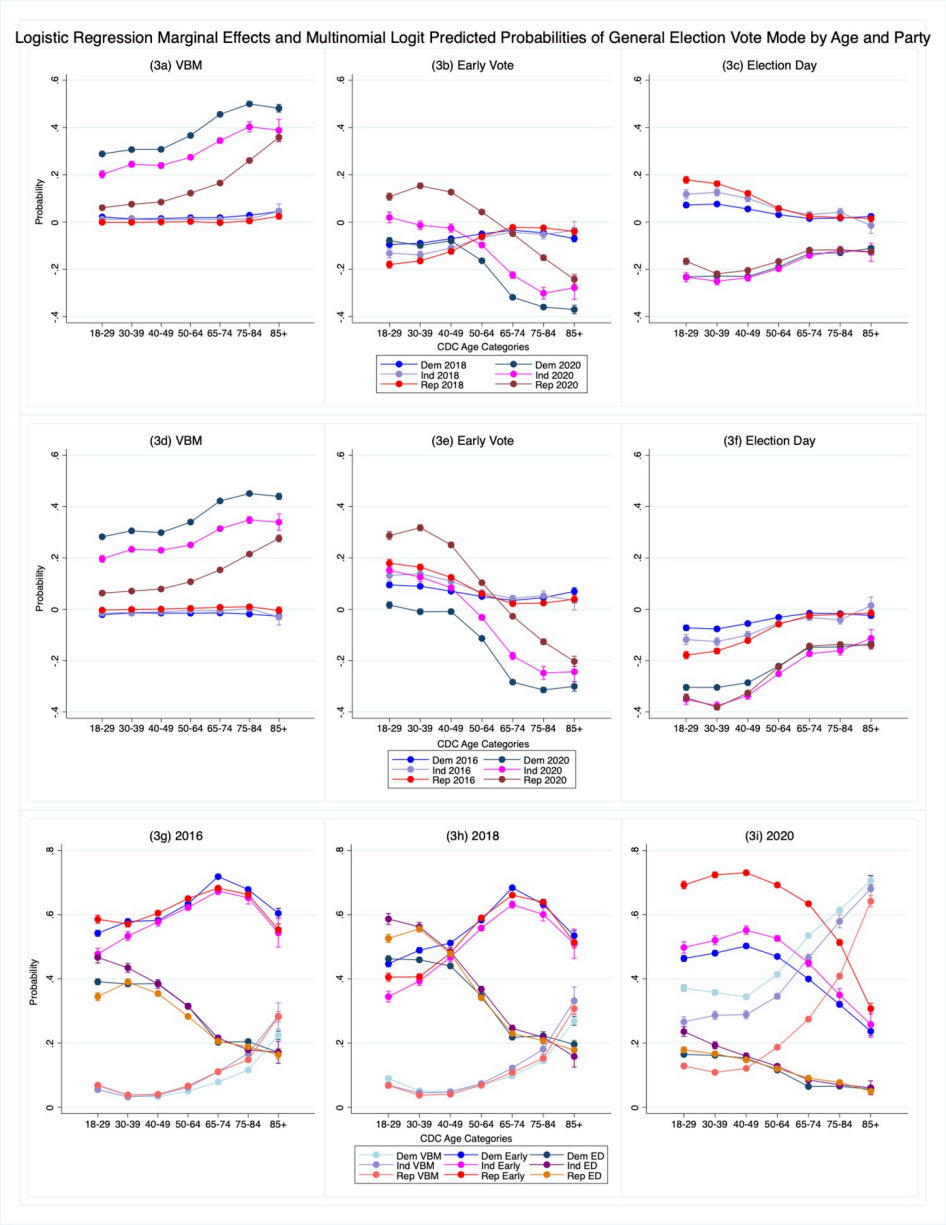
a–i. Logistic regression marginal effects and multinomial logit predicted probabilities of general election vote mode by age and party.

The first six panels in Fig 3 present visualizations of the difference in the probability of vote mode choice in the pandemic context (election year) for different party and age combinations including 95% confidence intervals, generated from the models for each vote mode: VBM, early and Election Day. In Fig 3A–3C we estimate the marginal effect of 2020 and 2018 compared to 2016 for different categories of age and party. We also estimate the average difference between 2018 and 2020 and show those by vote mode in Fig 3D–3F. Finally, for a fuller understanding of the behavioral change, in Fig 3G–3I we present the election year multinomial logit predicted probabilities (as opposed to the difference-in-differences). Full model results can be found in S4–S12 Tables and descriptive information can be found in S1–S2 Tables. Taken as a whole, the results demonstrate how drastically voting changed during the pandemic and how both health risks and partisanship affected decision-making.

Fig 3A shows that the difference in the effects of party and age between 2016 and 2018 on VBM is quite small or nonexistent with all three marginal effects lines hovering around 0. This shows that, prior to 2020 and COVID-19, party and age effects regarding VBM were basically constant across general elections. Consequently, we can infer that partisan behavior was not a major factor in VBM choice in either 2016 or 2018 and not important substantively. In contrast the difference between 2016 and 2020 (Fig 3A) or 2018 and 2020 (Fig 3D) is large and shows both partisan and age effects suggesting that both health risk and polarized elite messaging around vote-mode risk drove vote mode choices.

The results show support for a number of our hypotheses. While all voters are more likely to VBM than in previous years, consistent with hypothesis 1, Democrats were more likely to VBM than either Independents or Republicans, consistent with hypothesis 3a, and Independents were more likely to VBM than Republicans consistent with hypothesis 3b. Moreover, older voters among all partisan groups are more likely to VBM than their younger counterparts consistent with hypothesis 2. The effects are large. The youngest Democrats aged 18–49 were about three times more likely to VBM in 2020 than in 2016 (Fig 3A), while Democrats aged 65+ were, on average, about 4.5 times more likely to VBM. Republicans were significantly more likely to VBM in 2020, but the effects were smaller especially for younger voters aged 18–49 averaging about a .075 increase, while for the oldest voters 85+ the difference was about .28 more. Interestingly the oldest partisans are the least far apart on selecting VBM suggesting that they were motivated to vote most similarly, which is consistent with our hypotheses about the relationship between health risk, defined as age, and VBM. These results are mirrored when we look at Fig 3D that shows the results of the models for 2018–2020.

Fig 3B shows the differences in age and party between 2016 and 2018 and 2016 and 2020 for in-person early voters, and 3e for 2018 and 2020. Once again, we see mostly overlapping party results for 2016 and 2018 with older voters especially hovering close to 0 and therefore were about equally as likely to vote early in 2016 as they were in 2018. Younger voters were slightly less likely to vote early in 2018 than in 2016, which likely reflects the move to a midterm election and consequently less campaign mobilization. This is also supported by the results in Fig 3C which show that Election Day voting slightly increases for these same voters. Most importantly, the similarities in lines suggests party mattered little in vote mode decisions in 2018.

However, when we examine the difference between 2016 and 2020 (3b) or 2018 and 2020 (3e) we see strong party and age effects. Republicans were more likely to vote in-person early than Democrats or Independents consistent with hypothesis 3c. We also see that younger Republicans and Independents were more likely to vote in-person early than in 2016 or 2018, but as voters age across all groups they are less likely to vote in-person early because they are more likely to VBM (consistent with hypothesis 1 and hypothesis 2).

Fig 3C and 3F show the difference for in-person Election Day voting. The top three lines in Fig 3C show the change in the predicted probability from 2016 to 2018. The lines show that younger partisans, especially Republicans, were more likely to vote on Election Day in 2018 than in 2016. Again, this difference is likely due to changes in campaign mobilization patterns in a deep blue state from a presidential to a midterm election. But for the most part, the party lines are overlapping and close to zero, especially for older and more habitual voters, suggesting that Election Day voters in 2016 had about the same likelihood of Election Day voting as they did in 2018. The difference between 2016 and 2020, however, shows that partisans across age groups were equally, but less likely to vote on Election Day. All voters abandoned Election Day equally and based upon Fig 3A and 3B instead most Democrats and Republicans selected vote modes consistent with the government’s messaging regarding health risk, and party messaging regarding vote-count risks, with Democrats selecting mainly VBM and especially younger Republicans selecting mainly early voting, while older Republicans were more likely to VBM.

We also show in Fig 3G–3I predicted probabilities of vote modes for each year from the multinomial logit models, as opposed to the difference in predicted probabilities, using the same panel data. These figures provide context and enhance our understanding of vote mode decisions over time the and the different relationships we observe in Fig 3A–3C. Similar to Fig 3A–3F, these shows that in both 2016 and 2018 partisans made very similar decisions around their vote mode with only small variations that represent changes in election context and differences in party and campaign mobilization efforts [38]. But in 2020 both age and party mobilized voters differently. Compared to the tight clusters of lines across party we saw in Fig 3G–3I looks like chaos with lots of group variation.

**Fig 4:**
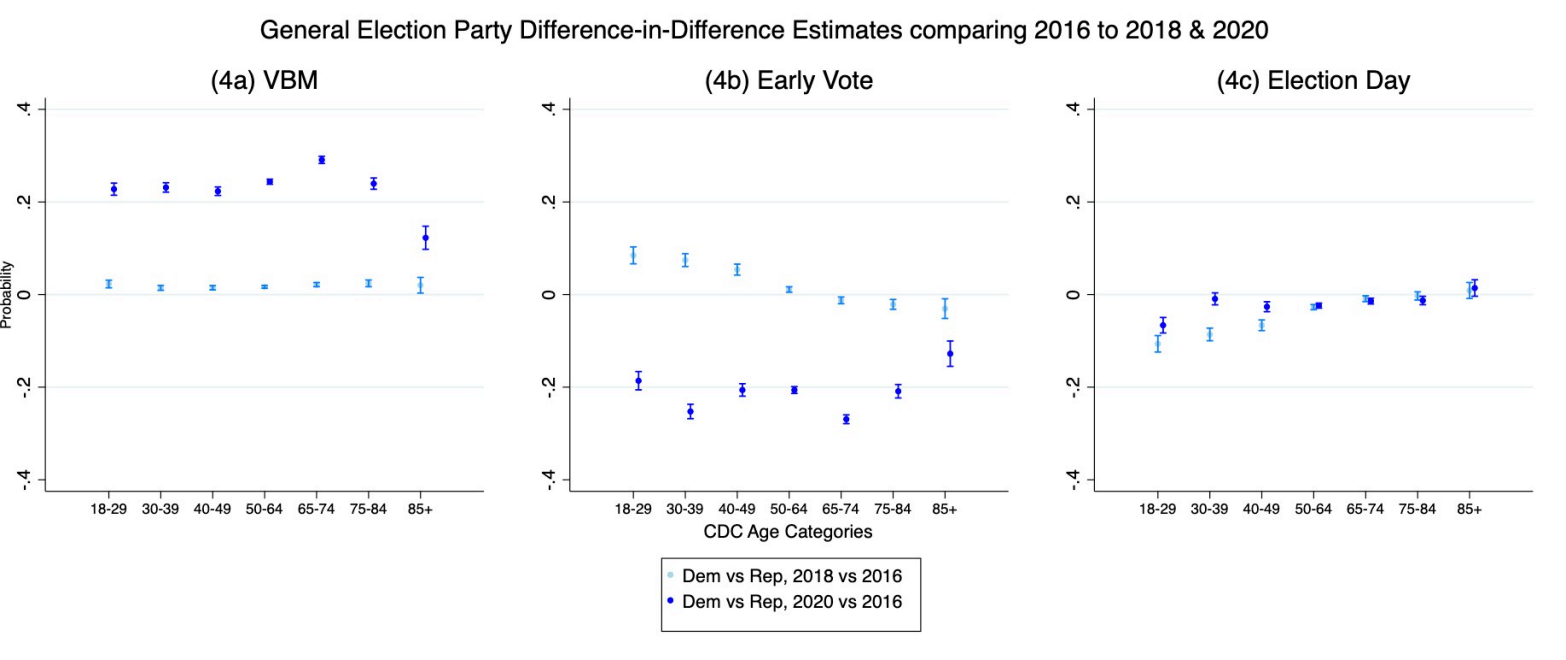
a-c. Party difference-in-differences estimates.

In Fig 4 we test hypothesis 3d by estimating whether the difference in treatment effects (effect of age category in 2020 compared to 2016) varies by party. Based on our argument, we should find statistically and substantively significant differences between parties across the 2020 and 2016 elections. For purposes of clarity, we focus only on Democrats versus Republicans where our predictions are the strongest. The point estimates compare the party difference (Democrat versus Republican) in the average marginal effect of the election context within each age category. In all the Figures, the zero line on the Y axis indicates no difference in the marginal effects across party. Positive values indicate the marginal effects between elections for Democrats exceeds Republicans, and negative values indicate the reverse. Based on our argument, we expect positive party difference-in-differences in VBM to be present throughout the age categories.

Fig 4A shows that in all age categories, the difference in the difference in the marginal effects across election years was greater for Democrats than Republicans, and greatest in the age-risk category, 65–74. The party difference-in-differences declines for the top age category, but this is because Republicans voted by mail at increasing rates as age increased while Democrats had a higher baseline and leveled off after an initial increase (see Fig 3A). In Fig 4B, we continue to see significant difference-in-differences between parties, but this time, the election year effects on early voting in each age category is greater for Republicans than Democrats, with the greatest gap occurring in the 65–74 age category. Fig 4C shows only slight or no differences across parties, and where differences exist, they are in the predicted direction. All three figures include a comparison to the party difference-in-differences for 2018 to 2016. These show that the partisan gaps occur in each year, but they are substantively smaller and hover around zero. This suggests that the pandemic had quite different effects than typical differences that occur from party and campaign related mobilization effects.

In the Supplemental Information section S1 Fig shows the graphs for the cross-sectional model for VBM, early and Election Day voting. The results are substantively similar and show that our results are robust if we estimate our models using the entire electorate in each year.

### Primary election vote mode

To validate our models further, we turn to the primary election held on Tuesday, June 2, 2020. In response to the pandemic, 27 NM county clerks petitioned the NM Supreme Court to allow them to conduct an all-mail election of all eligible electors [39]. The NM GOP sued claiming such changes were against NM law and questioned the reliability of mail balloting in NM. The NM Supreme Court agreed with the NM GOP and charged the county clerks with instead mailing out a request for a VBM application to each eligible elector.

**Fig 5:**
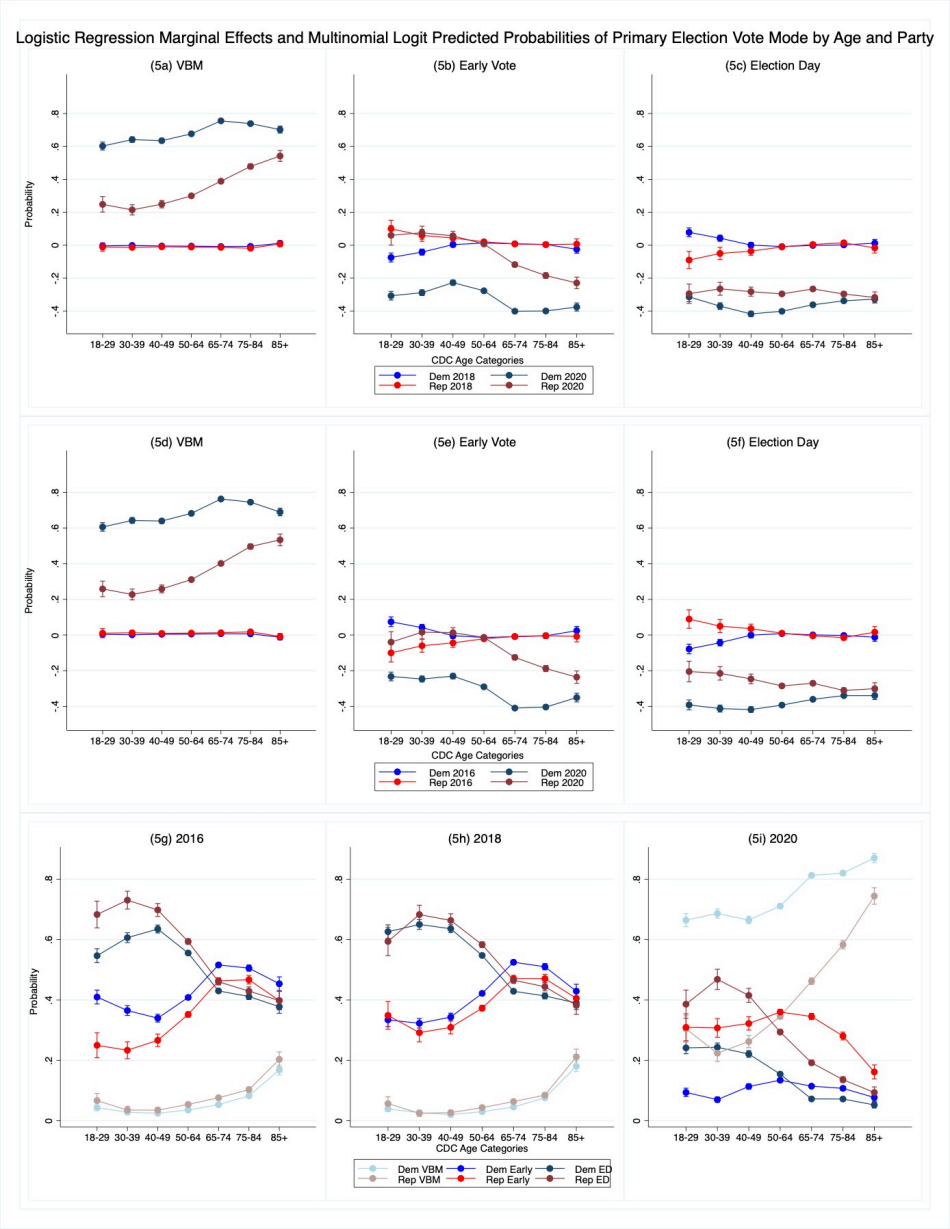
a–i. Logistic regression marginal effects and multinomial logit predicted probabilities of primary election vote mode by age and party.

Like our design above, we present multiple figures highlighting how both age and party influenced VBM decisions in the party preference primary. Fig 5A–5C present the difference in the marginal effects of vote mode choice for 2020 in different age and party categories compared to the same groups in the baseline of 2016. Fig 5D–5F show the same results for when the base category is 2018. Fig 5G–5I present the multinomial logits for each year of the predicted probabilities for vote mode to provide context to the difference-in-differences graphs on the rates of voting mode across all 3 years.

First, notice that the placebo model, comparing 2016 to 2018, shows no difference between 2016 and 2018 for VBM as the line hovers around zero for both parties. The multinomial logits, Fig 5G and 5H, also show us that there was very little VBM in either primary contest. In contrast, however, we see a big difference between 2016 and 2020, comparing presidential election to presidential election in Fig 5A, and a big difference between 2018 and 2020, comparing the midterm to the presidential in Fig 5D. The results show that both Democrats and Republicans regardless of age were more likely to VBM in the primary in 2020, consistent with hypothesis 1. We also find that there is a strong VBM effect by party that averages about a .35 difference for the youngest voters and for those over 85 about a .125 difference with Democrats overall more likely to VBM in 2020 than Republicans consistent with hypothesis 3a.

Fig 5B shows the change in early voting from 2016 to 2018 and 2016 to 2020 and Fig 5E show the same change for 2018 and 2020 and 2018 to 2016, the latter comparison being just the reverse of 2016 to 2018. We see a bit larger difference between the 2016 and 2018 primary than we did for the general where they were virtually the same. This is not surprising given that the 2016 presidential preference primary had not been decided for Democrats and there was a lot of enthusiasm among Republicans for their nominee and thus we would expect mobilization patterns to be stronger in 2016 than in 2018, which is what we see in Fig 5B.

The results show that in 2018 Democrats and Republicans average in-person early vote hovered close to zero showing that patterns of voting were very similar across elections. When we consider the change from 2016 we see a party gap, consistent with hypothesis 3a, with the Republican probability of voting early being higher than the Democrats, consistent with hypothesis 3c. The youngest Republicans who voted early in 2020 were about as likely to do as they were in 2016 or 2018, while Republican over 65 were significantly less likely to vote early by bout .2 and Democrats by .4. There is also a clear partisan gap in 2020 compared to 2018 and 2016 with an average difference around .2 for younger Democratic voters, but reduces to about .1 for the oldest voters who are the most at risk for COVID-19 complications. This is consistent with the risk hypothesis 3d that younger voters should be more likely to choose an in-person option over VBM.

Fig 5C shows the change in Election Day voting from 2016 to 2018 and 2016 to 2020 and Fig 5F shows the same change for 2018 and 2020. There are differences between Democrats and Republicans when Election Day results are compared, but they are relatively small and close to 0 suggesting vote mode was more similar than different across these elections. This is comparable to what we saw in Fig 3C and 3F. Younger Republicans are slightly less likely to vote on Election Day than their counterparts in 2016, but the presidential preference primary likely mobilized more of these voters into an earlier voting mode. The more interesting results in these figures are comparing 2016 and 2020 and comparing 2018 and 2020. These results show Election Day voting is much less likely in 2020. Comparing 2016 with 2020 shows that Republicans are on average about .3 less likely to vote on Election Day and Democrats are on average almost .4 less likely to vote on Election Day than in 2016, and we see a similar pattern with 2018 and 2020.

Finally, we show the multinomial logits for individual years in the last row of figures for context. As in the general election graphs (Fig 3G–3I), the primary data show tremendous stability in vote mode choice between 2016, Fig 5G, and 2018, Fig 5H, and virtually no party differences. There are some age effects. We see that typically older voters are on average about .1 to .2 more likely to VBM which is why we often see the oldest voters change in probability in 2020 to drop a little bit compared to the next oldest voters.

In summary, the primary results support the results we see in the general election. More voters VBM, Democrats more so than Republicans. Republicans were more likely to vote early than Democrats and there were strong age effects for VBM with older voters more likely to choose this option than younger voters.

We also show in the Supplemental Information S2 Fig the cross-sectional results for the entire population of primary voters. The cross-sectional primary results tell the same story as the panel and help us to demonstrate that it is the election year and not just these voters that responded to the pandemic and its partisan messages.

**Fig 6:**
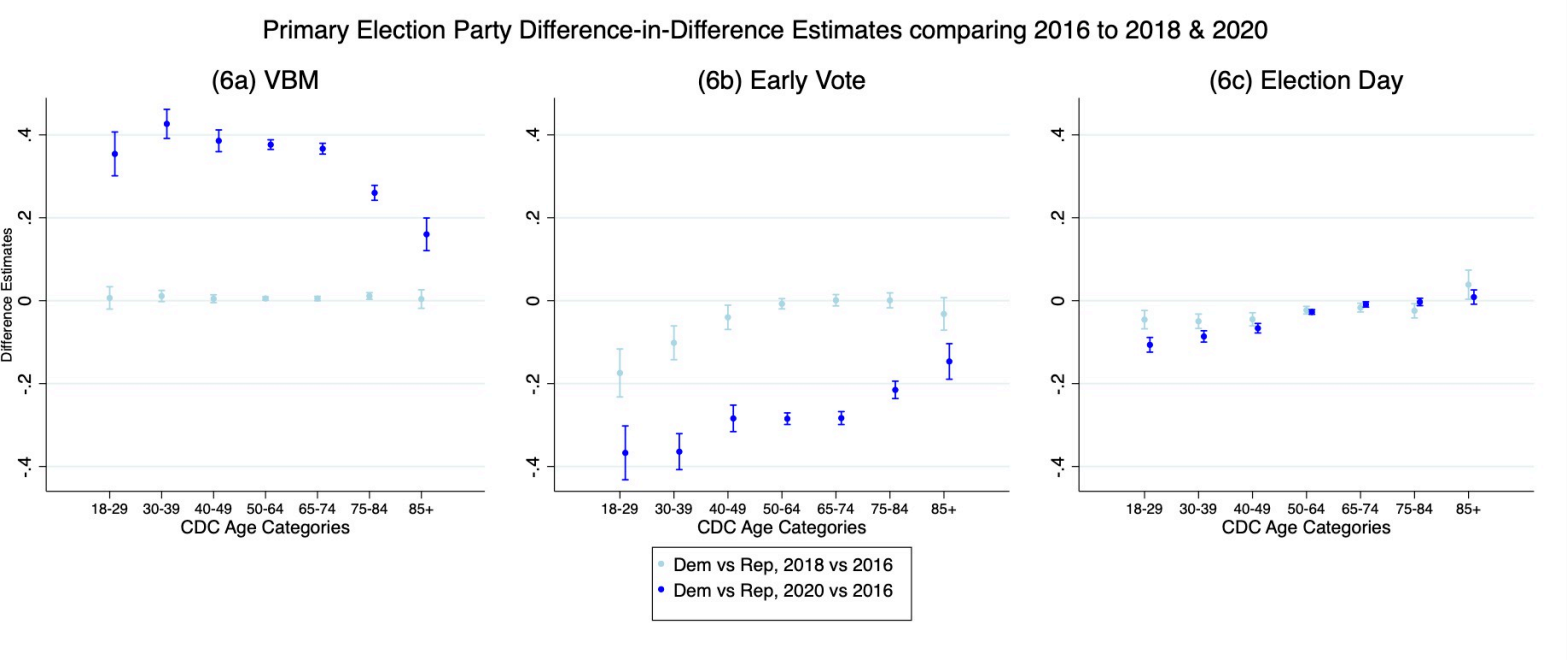
a-c. Primary election party difference-in-differences.

In Fig 6, like Fig 4, we explore the party difference-in-differences to determine whether there were partisan differences in the average marginal effect election context across age categories. Like in the general election panel data, we find in 6a a significant and substantively large partisan gap in the effect of age in the primary VBM. The declining difference-in-differences across age categories shows that even though party moderates the age effect, the gap in the average marginal effect between elections for Democrats and Republicans becomes significantly smaller for older voters. The party moderation of the pandemic effect is greatest for voters who face the least risk from COVID-19, and smallest for older voters who face the most risk. In Fig 6B, we see a reversed pattern with the average marginal effect of the election context for each age category greater for Republicans than Democrats. The party difference-in-differences becomes much smaller among older voters as both groups are more similar in their vote mode selection. Few differences emerge between parties in primary Election Day voting, but compared to the effects in other modes, the difference-in-differences is substantively small and, for some age categories, statistically insignificant. The party difference in the conditional marginal effect by age in 2018–2016 also shows partisan effects for some age categories, but the effects are much smaller in magnitude than the 2020–2016 difference. The differences we see pre-pandemic reflect differences in party mobilization which are much smaller than the differences we see once risk and party become proxies for different levels of perceived risk during the pandemic.

### Timing of early voting

Hypothesis 4a suggests that voters should vote earlier in the process in 2020 in response to messages from elites and government institutions like the CDC that encouraged it. We already saw in Fig 3 that especially younger Republicans and Independents increased their early voting relative to 2016 or 2018, but here we specifically ask, did in-person voters choose to vote, on average, earlier in the process than they typically do to reduce the density of voters in vote centers?

This portion of our analysis focuses on the panel of voters who voted in person in both 2018 and 2020. We calculated the dependent variable as the number of days from the day before the start of in-person early voting, coded 0, to Election Day, coded 29. Recall, NM’s early in-person voting system begins 28 days before the general election in the County Clerk’s Office. At 21 days before the election this process expands to a larger number of locations across each county. The last day of early voting is the Saturday before Election Day.

**Fig 7:**
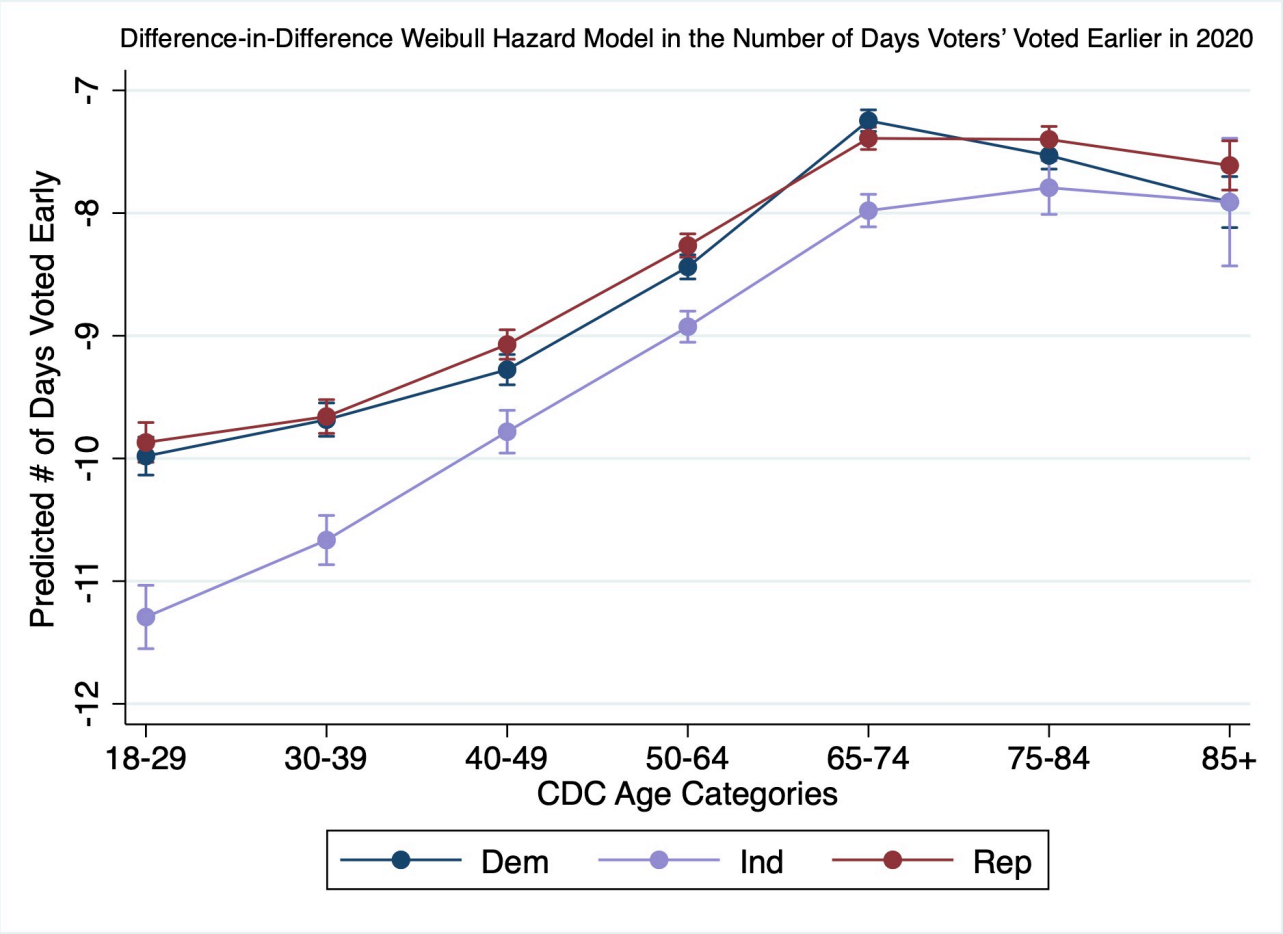
Difference-in-differences Weibull hazard model in the number of days voters’ voted earlier in 2020.

To obtain better causal estimates, Fig 7 illustrates the difference in the average number of days those early voters waited (1 to 28) to vote once early voting opened in the state. Using a survival model with a Weibull distribution and accelerated failure time, we compare 2018 to 2020 to show the difference in the average number of days those voters voted later. Consistent with our previous models, we interact age and party and graph the results. Because this is a survival model, we are counting from the beginning of early voting. Thus, Fig 7 shows that the youngest Independents survived, on average, 11.5 days longer in 2018 than 2020, i.e., they voted 11.5 days closer to Election Day. Older Independents voted about 8 days closer to Election Day in 2018 compared to 2020. Model parameters can be found in S16 Table.

The results in Fig 7 clearly show that voters responded to elite messaging about when to vote consistent with hypothesis 4a. Across all age categories, voters in 2018 compared to 2020 survived longer, or voted later in the process, that is they voted closer to Election Day in 2018 than in 2020. Younger partisan voters aged 18–49 voted between 9 and 10 days later in 2018 than in 2020 and younger independents voted on average about 1 day later than their partisan counterparts. Older voters over 65 voted between 7 and 8 days later in 2018. This means that older voters were more likely to “fail” or vote further from Election Day than younger voters consistent with hypothesis 4b. Thus, older voters tended to vote 2 or 3 days earlier in the process than younger voters.

We also find that the average difference in the number of days is not substantively different for Democrats and Republicans. Thus, while we saw in our earlier analysis that there was partisan polarization around voting mode, once Democrats and Republicans chose to vote early their behavior about when to vote was similar. Younger independents were likely to fail by a little over a day later, but older independents were a lot closer to their partisan counterparts consistent with our risk hypothesis. Therefore, these results support our hypothesis that messaging regarding earlier voting around a need for a reduction in polling place density strongly influenced when voters decided to vote and influenced them more similarly regardless of partisanship. Since both party elites agreed that de-densifying the polling place was a good idea, no party polarization was forthcoming.

## Discussion

We examined voter administrative data in NM to understand vote mode decision-making during the pandemic. Administrative data are very reliable and valid and, unlike survey responses, prevent voters from engaging in socially desirable answers about their behavior and choices. These factors along with our ability to create a panel of voters that compare 2020 election data with the two prior general and primary elections with nearly identical vote conditions provides strong internal and external validity for making causal inferences.

NM is an excellent case to make this comparison because it did not change its election environment in 2020, which allows us to make comparisons over time, and it has an open election environment that allows voters a great deal of choice. We expect that these results would be transferable to other “no excuse” VBM states that maintained or expanded all 3 voting modes. However, we would not expect these results to inform our understanding of vote mode changes in states that are already universal VBM states or became universal VBM states because of the pandemic.

From the start of the pandemic, age was a clear risk-factor for severe illness and death. Therefore, we expected and found that age-related health risks shaped choice of vote mode, with younger and older voters making quite different voting decisions. While all voters were more likely to VBM than in the previous 2 general elections, younger voters were more likely to vote early in person than older voters who were more likely to VBM, and in person older voters were more likely to choose to vote earlier in the process than younger voters.

Importantly, our results offer crucial nuance about how heterogeneous and polarized information environments can shape voting choices. The results across the primary and general elections show concrete evidence of partisan polarized choices in voting behavior, even among individuals facing similar levels of risk based upon age from COVID-19. In comparison to the placebo comparisons of 2016 and 2018, where Republicans and Democrats selected similar vote modes, 2020 looks very different, with partisans selecting very different routes to cast their ballots. Top Republican leaders emphasized the risks of miscounts, errors, and fraud in VBM, while top Democratic leaders emphasized confidence in VBM and the reduction in health-risk from social distancing. Together, these different causal narratives about VBM integrity provided powerful incentives for Republicans to vote early in person at greater rates than Democrats and Independents. In this environment, Republicans opted more for a risk-balanced strategy by selecting a vote mode that was somewhat riskier for their health than VBM, but less risky than potentially dense and long waits on Election Day. In-person voting posed no risk for a lost vote since voters could directly deposit their ballot into the voting machine for counting rather than rely on third parties to deliver their vote to the county clerk for acceptance and counting.

More generally, these results highlight how catastrophic events, like a pandemic, can result in complex and competing risks in the minds of the public as they strive to manage day-to-day choices that used to be routine. Catastrophes encourage both attentiveness to risk information and risk-averting behavior. However, partisan and polarized information flows can result in very different calculations, even if individuals are using similar decision processes. While vote mode was largely a non-partisan issue prior to 2020, vote mode was highly partisan in 2020 and partisan voters mitigated their risks in different ways depending on whether they were voting in person or by mail in keeping with information from both the CDC and other elites. Importantly, the decision process that gives rise to vote mode choice is likely at play in many settings. Polarized choices by age and party around vaccines, engaging in social distancing, and mask use have been shown to operate similarly in nature.

For example, younger citizens who are less susceptible to COVID-19 complications and Republicans who consistently appear less concerned about threats from the virus are less likely to have taken the vaccine [40, 41]. When we look at charts of the relationship between the COVID-19 vaccine and age, we find that older populations are much more likely to obtain the vaccine than younger populations and that the results are largely monotonic and comparable to what we see with vote mode [41].

Survey data also suggest that partisanship is playing a large role in COVID-19 behaviors with 41% of all Republicans or Republican leaning citizens indicating they are unvaccinated compared to 32% of independents and only 9% of Democrats [40]. Other research shows that Republicans seek out information about COVID-19 and its severity less frequently than Democrats [42]. Even after controlling for key demographic factors, the partisan gap in beliefs about the severity of COVID-19 and the importance of social distancing remain strong [43]. Overall, the picture becomes clear that partisan polarization has vastly manifested itself in the behavioral choices of Republicans and Democrats related to COVID-19.

Our data suggest that the differences in behavior noted above are likely due to estimates of health-related risks that are amplified within heterogeneous and partisan information environments. Therefore, our study of vote mode, where we have accurate comparisons to behavior prior to the pandemic, gives us the clearest evidence of the nuanced ways complex risk information becomes translated into behavioral choices. Therefore, the use of high-quality observational data in the form of administrative data in our study aids in corroborating and in offering very precise estimates compared to the survey data regarding the health risks and partisan choices related to voting in the 2020 election.

## Supporting information

S1 Table Panel general election descriptive statistics. (PDF)

S2 Table Panel primary election descriptive statistics. (PDF)

S3 Table Hazard model descriptive statistics. (PDF)

S4 Table Logistic regression vote by mail general election with 2020 as base year (Fig 3A). (PDF)

S5 Table Logistic regression early vote general election with 2020 as base year (Fig 3B). (PDF)

S6 Table Logistic regression election day general election with 2020 as base year (Fig 3C). (PDF)

S7 Table Multinomial logistic regression vote mode general election 2016 (Fig 3G). (PDF)

S8 Table Multinomial logistic regression vote mode general election 2018 (Fig 3H). (PDF)

S9 Table Multinomial logistic regression vote mode general election 2020 (Fig 3I). (PDF)

S10 Table Logistic regression vote by mail primary election with 2020 as base year (Fig 5A). (PDF)

S11 Table Logistic regression early vote primary election with 2020 as base year (Fig 5B). (PDF)

S12 Table Logistic regression election day primary with 2020 as base year (Fig 5C). (PDF)

S13 Table Multinomial logistic regression vote mode primary election 2016 (Fig 5G). (PDF)

S14 Table Multinomial logistic regression vote mode primary election 2018 (Fig 5H). (PDF)

S15 Table Multinomial logistic regression vote mode primary election 2020 (Fig 5I). (PDF)

S16 Table Hazard model comparing likelihood to vote early by day across 2018 and 2020 (Fig 7). (PDF)

S1 Fig Logistic regression marginal effects and multinomial logit predicted probabilities of cross-sectional general election vote mode by age and party 3a – 3i graphs. (TIF)

S2 Fig Logistic regression marginal effects and multinomial logit predicted probabilities of primary election vote mode by age and party 5a – 5i graph. (TIF)
