# Supplementary material for: Should I vote-by-mail or in person? The impact of COVID-19 risk factors and partisanship on vote mode decisions in the 2020 presidential election
Source: PLoS One. 2022 Sep 15;17(9):e0274357. doi: 10.1371/journal.pone.0274357 (PMC9477279; doi:10.1371/journal.pone.0274357)
Supplement: S1 Table — (PDF) [file pone.0274357.s001.pdf]

S1 Table. Panel General Election Descriptive Statistics

Variable	N	Mean	Std. Dev.	Min	Max
Voted by Mail	937416	0.173	0.378	0.000	1.000
Voted Early	937416	0.578	0.494	0.000	1.000
Voted on Election Day	937416	0.249	0.432	0.000	1.000
Age Category: 18-24 y/o	937416	0.062	0.242	0.000	1.000
Age Category: 25-29 y/o	937416	0.095	0.293	0.000	1.000
Age Category: 30-39 y/o	937416	0.118	0.323	0.000	1.000
Age Category: 40-49 y/o	937416	0.380	0.485	0.000	1.000
Age Category: 50-64 y/o	937416	0.226	0.418	0.000	1.000
Age Category: 65-74 y/o	937416	0.095	0.293	0.000	1.000
Age Category: 75-84 y/o	937416	0.024	0.152	0.000	1.000
Age Category: 85+ y/o	937416	0.523	0.499	0.000	1.000
Democrats	937416	0.113	0.317	0.000	1.000
Independents	937416	0.364	0.481	0.000	1.000
Republicans	937416	0.315	0.464	0.000	1.000
Hispanic	937416	0.007	0.084	0.000	1.000
Asian	937416	0.008	0.088	0.000	1.000
Black	937416	0.032	0.175	0.000	1.000
Other Race/Ethnicity	937412	0.549	0.498	0.000	1.000
Female	937412	0.000	0.013	0.000	1.000
Other Sex	937416	0.173	0.378	0.000	1.000
Bernalillo	937416	0.353	0.478	0.000	1.000
Catron	937416	0.003	0.050	0.000	1.000
Chaves	937416	0.025	0.155	0.000	1.000
Cibola	937416	0.010	0.099	0.000	1.000
Colfax	937416	0.007	0.082	0.000	1.000
Curry	937416	0.015	0.121	0.000	1.000
De Baca	937416	0.001	0.035	0.000	1.000
Dona Ana	937416	0.084	0.277	0.000	1.000
Eddy	937416	0.023	0.149	0.000	1.000
Grant	937416	0.017	0.128	0.000	1.000
Guadalupe	937416	0.003	0.050	0.000	1.000
Harding	937416	0.001	0.026	0.000	1.000
Hidalgo	937416	0.002	0.048	0.000	1.000
Lea	937416	0.020	0.139	0.000	1.000
Lincoln	937416	0.011	0.105	0.000	1.000
Los Alamos	937416	0.014	0.118	0.000	1.000
Luna	937416	0.009	0.092	0.000	1.000
McKinley	937416	0.025	0.156	0.000	1.000
Mora	937416	0.003	0.059	0.000	1.000
Otero	937416	0.024	0.154	0.000	1.000
Quay	937416	0.004	0.065	0.000	1.000
Rio Arriba	937416	0.020	0.139	0.000	1.000
Roosevelt	937416	0.007	0.082	0.000	1.000
San Juan	937416	0.056	0.230	0.000	1.000
San Miguel	937416	0.014	0.117	0.000	1.000
Sandoval	937416	0.077	0.266	0.000	1.000
Santa Fe	937416	0.094	0.291	0.000	1.000

Sierra	937416	0.006	0.079	0.000	1.000
Socorro	937416	0.009	0.092	0.000	1.000
Taos	937416	0.020	0.142	0.000	1.000
Torrance	937416	0.008	0.087	0.000	1.000
Union	937416	0.002	0.046	0.000	1.000
Valencia	937416	0.036	0.186	0.000	1.000
