# Supplementary material for: Should I vote-by-mail or in person? The impact of COVID-19 risk factors and partisanship on vote mode decisions in the 2020 presidential election
Source: PLoS One. 2022 Sep 15;17(9):e0274357. doi: 10.1371/journal.pone.0274357 (PMC9477279; doi:10.1371/journal.pone.0274357)
Supplement: S2 Table — (PDF) [file pone.0274357.s002.pdf]

S2 Table. Panel Primary Election Descriptive Statistics

Variable	N	Mean	Std. Dev.	Min	Max
Voted by Mail	269370	0.406	0.491	0.000	1.000
Voted Early	269370	0.352	0.478	0.000	1.000
Voted on Election Day	269370	0.393	0.488	0.000	1.000
Age Category: 18-29 y/o	269370	0.023	0.151	0.000	1.000
Age Category: 30-39 y/o	269370	0.046	0.209	0.000	1.000
Age Category: 40-49 y/o	269370	0.074	0.261	0.000	1.000
Age Category: 50-64 y/o	269370	0.367	0.482	0.000	1.000
Age Category: 65-74 y/o	269370	0.314	0.464	0.000	1.000
Age Category: 75-84 y/o	269370	0.146	0.353	0.000	1.000
Age Category: 85+ y/o	269370	0.030	0.172	0.000	1.000
Democrats	269370	0.703	0.457	0.000	1.000
Republicans	269370	0.297	0.457	0.000	1.000
Hispanic	269370	0.326	0.469	0.000	1.000
Asian	269370	0.004	0.066	0.000	1.000
Black	269370	0.008	0.087	0.000	1.000
Other Race/Ethnicity	269370	0.027	0.163	0.000	1.000
Female	269369	0.571	0.495	0.000	1.000
Other Sex	269369	0.000	0.012	0.000	1.000
Bernalillo	269370	0.331	0.471	0.000	1.000
Catron	269370	0.004	0.060	0.000	1.000
Chaves	269370	0.024	0.153	0.000	1.000
Cibola	269370	0.011	0.103	0.000	1.000
Colfax	269370	0.008	0.091	0.000	1.000
Curry	269370	0.012	0.109	0.000	1.000
De Baca	269370	0.001	0.037	0.000	1.000
Dona Ana	269370	0.062	0.241	0.000	1.000
Eddy	269370	0.018	0.133	0.000	1.000
Grant	269370	0.024	0.152	0.000	1.000
Guadalupe	269370	0.004	0.063	0.000	1.000
Harding	269370	0.001	0.035	0.000	1.000
Hidalgo	269370	0.002	0.048	0.000	1.000
Lea	269370	0.017	0.128	0.000	1.000
Lincoln	269370	0.010	0.101	0.000	1.000
Los Alamos	269370	0.014	0.119	0.000	1.000
Luna	269370	0.010	0.097	0.000	1.000
McKinley	269370	0.025	0.157	0.000	1.000
Mora	269370	0.007	0.086	0.000	1.000
Otero	269370	0.024	0.152	0.000	1.000
Quay	269370	0.006	0.074	0.000	1.000
Rio Arriba	269370	0.034	0.180	0.000	1.000
Roosevelt	269370	0.007	0.082	0.000	1.000
San Juan	269370	0.045	0.207	0.000	1.000
San Miguel	269370	0.023	0.150	0.000	1.000
Sandoval	269370	0.067	0.250	0.000	1.000
Santa Fe	269370	0.119	0.324	0.000	1.000
Sierra	269370	0.007	0.084	0.000	1.000
Socorro	269370	0.011	0.104	0.000	1.000

Taos	269370	0.029	0.168	0.000	1.000
Torrance	269370	0.009	0.093	0.000	1.000
Union	269370	0.003	0.051	0.000	1.000
Valencia	269370	0.032	0.175	0.000	1.000
