# Supplementary material for: Should I vote-by-mail or in person? The impact of COVID-19 risk factors and partisanship on vote mode decisions in the 2020 presidential election
Source: PLoS One. 2022 Sep 15;17(9):e0274357. doi: 10.1371/journal.pone.0274357 (PMC9477279; doi:10.1371/journal.pone.0274357)
Supplement: S3 Table — (PDF) [file pone.0274357.s003.pdf]

S3 Table. Hazard Model Descriptive Statistics

Variable	N	Mean	Std. Dev.	Min	Max
Voted Early	485185	.706	.455	0	1
Voted on Election Day	485185	.294	.455	0	1
Days Since Voting Opens	485185	20.518	7.653	1	29
Age Category: 18-29 y/o	485185	.066	.247	0	1
Age Category: 30-39 y/o	485185	.103	.304	0	1
Age Category: 40-49 y/o	485185	.129	.336	0	1
Age Category: 50-64 y/o	485185	.394	.489	0	1
Age Category: 65-74 y/o	485185	.212	.408	0	1
Age Category: 75-84 y/o	485185	.081	.272	0	1
Age Category: 85+ y/o	485185	.016	.124	0	1
Democrats	485185	.491	.5	0	1
Independents	485185	.113	.317	0	1
Republicans	485185	.396	.489	0	1
Hispanic	485185	.316	.465	0	1
Asian	485185	.007	.081	0	1
Black	485185	.008	.088	0	1
Other Race/Ethnicity	485185	.035	.183	0	1
Female	485185	.539	.498	0	1
Other Sex	485185	0	.012	0	1
