# Supplementary material for: Should I vote-by-mail or in person? The impact of COVID-19 risk factors and partisanship on vote mode decisions in the 2020 presidential election
Source: PLoS One. 2022 Sep 15;17(9):e0274357. doi: 10.1371/journal.pone.0274357 (PMC9477279; doi:10.1371/journal.pone.0274357)
Supplement: S4 Table — (PDF) [file pone.0274357.s004.pdf]

S4 Table. Logistic Regression Vote by Mail General Election with 2020 as Base Year (Fig 3a)

	Coef.	SE	t-value	p-value	[95% Conf	Interval]	Sig
Age Categories							
30-39 y/o	-.618	.059	-10.52	0	-.733	-.503	***
40-49 y/o	-.693	.057	-12.11	0	-.805	-.581	***
50-64 y/o	-.194	.044	-4.45	0	-.28	-.109	***
65-74 y/o	.271	.044	6.19	0	.185	.357	***
75-84 y/o	.731	.047	15.66	0	.64	.823	***
85+ y/o	1.514	.055	27.45	0	1.406	1.622	***
Political Party							
Independent	-.174	.088	-1.96	.05	-.347	0	**
Republican	.152	.065	2.33	.02	.024	.281	**
Election Year							
2018	.332	.037	8.98	0	.26	.405	***
2020	2.158	.041	53.08	0	2.078	2.238	***
Party X Year							
Ind X 2018	-.101	.085	-1.19	.235	-.266	.065	
Ind X 2020	-.302	.091	-3.33	.001	-.479	-.124	***
Rep X 2018	-.346	.064	-5.38	0	-.472	-.22	***
Rep X 2020	-1.458	.07	-20.97	0	-1.594	-1.322	***
Age X Year							
30-39 X 2018	.018	.057	0.31	.757	-.095	.13	
30-39 X 2020	.577	.06	9.61	0	.459	.694	***
40-49 X 2018	.073	.054	1.34	.181	-.034	.179	
40-49 X 2020	.645	.058	11.05	0	.53	.759	***
50-64 X 2018	.001	.041	0.03	.973	-.079	.082	
50-64 X 2020	.494	.045	10.98	0	.406	.582	***
65-74 X 2018	-.093	.041	-2.26	.024	-.173	-.012	**
65-74 X 2020	.525	.045	11.62	0	.437	.614	***
75-84 X 2018	-.077	.043	-1.80	.072	-.161	.007	*
75-84 X 2020	.43	.049	8.80	0	.334	.525	***
85+ X 2018	-.083	.049	-1.67	.095	-.18	.014	*
85+ X 2020	.061	.061	0.99	.321	-.059	.181	
Age X Party							
30-39 X Ind	.053	.128	0.41	.681	-.198	.303	
30-39 X Rep	.045	.095	0.47	.635	-.141	.231	
40-49 X Ind	.299	.122	2.45	.014	.06	.538	**
40-49 X Rep	.158	.089	1.77	.077	-.017	.332	*
50-64 X Ind	.337	.098	3.44	.001	.145	.529	***
50-64 X Rep	.151	.071	2.14	.032	.013	.289	**
65-74 X Ind	.512	.098	5.23	0	.321	.704	***
65-74 X Rep	.246	.071	3.46	.001	.107	.386	***
75-84 X Ind	.545	.108	5.03	0	.333	.757	***
75-84 X Rep	.11	.075	1.47	.142	-.037	.256	
85+ X Ind	.429	.143	3.01	.003	.149	.708	***
85+ X Rep	.143	.086	1.65	.099	-.027	.312	*
Party X Age X Year							
Ind X 30-39 X 2018	.093	.127	0.73	.464	-.156	.343	
Ind X 30-39 X 2020	.098	.13	0.76	.449	-.156	.353	
Ind X 40-49 X 2018	-.058	.118	-0.49	.626	-.289	.174	
Ind X 40-49 X 2020	-.138	.123	-1.11	.265	-.38	.104	
Ind X 50-64 X 2018	-.049	.093	-0.53	.596	-.232	.134	
Ind X 50-64 X 2020	-.236	.1	-2.35	.019	-.432	-.039	**
Ind X 65-74 X 2018	-.026	.092	-0.28	.78	-.207	.155	
Ind X 65-74 X 2020	-.39	.101	-3.87	0	-.587	-.192	***

Ind X 75-84 X 2018	-.059	.1	-0.59	.554	-.255	.137	
Ind X 75-84 X 2020	-.304	.113	-2.69	.007	-.526	-.082	***
Ind X 85+ X 2018	.081	.119	0.68	.499	-.153	.315	
Ind X 85+ X 2020	-.182	.154	-1.18	.239	-.484	.121	
Rep X 30-39 X 2018	-.027	.095	-0.28	.779	-.213	.16	
Rep X 30-39 X 2020	-.142	.099	-1.43	.153	-.336	.052	
Rep X 40-49 X 2018	-.053	.088	-0.60	.548	-.227	.12	
Rep X 40-49 X 2020	-.147	.094	-1.57	.116	-.331	.036	
Rep X 50-64 X 2018	.044	.069	0.64	.524	-.091	.179	
Rep X 50-64 X 2020	-.001	.075	-0.01	.994	-.147	.146	
Rep X 65-74 X 2018	.081	.069	1.17	.241	-.054	.217	
Rep X 65-74 X 2020	-.087	.075	-1.16	.246	-.235	.06	
Rep X 75-84 X 2018	.128	.071	1.79	.073	-.012	.268	*
Rep X 75-84 X 2020	.297	.08	3.73	0	.141	.453	***
Rep X 85+ X 2018	.221	.079	2.80	.005	.067	.376	***
Rep X 85+ X 2020	.831	.095	8.71	0	.644	1.018	***
Hispanic	-.154	.009	-17.37	0	-.171	-.136	***
Asian	.258	.042	6.14	0	.175	.34	***
Black	-.042	.044	-0.96	.336	-.128	.044	
Other Race	-.302	.025	-12.01	0	-.352	-.253	***
Female	.177	.008	22.87	0	.162	.192	***
Other Sex	-.056	.214	-0.26	.794	-.476	.364	
County							
Catron	-.434	.093	-4.69	0	-.615	-.253	***
Chaves	-1.104	.033	-33.16	0	-1.169	-1.039	***
Cibola	-.982	.047	-20.79	0	-1.075	-.89	***
Colfax	-.502	.046	-10.84	0	-.593	-.411	***
Curry	-1.436	.036	-39.35	0	-1.507	-1.364	***
De Baca	-1.173	.137	-8.57	0	-1.441	-.905	***
Dona Ana	-.388	.014	-27.38	0	-.416	-.361	***
Eddy	-1.014	.033	-30.56	0	-1.079	-.949	***
Grant	-.597	.033	-18.00	0	-.662	-.532	***
Guadalupe	.201	.083	2.42	.015	.038	.364	**
Harding	.389	.164	2.37	.018	.067	.711	**
Hidalgo	-.587	.089	-6.57	0	-.762	-.412	***
Lea	-1.019	.038	-27.04	0	-1.093	-.945	***
Lincoln	-.537	.041	-13.10	0	-.617	-.456	***
Los Alamos	-.409	.03	-13.53	0	-.468	-.35	***
Luna	-.737	.045	-16.45	0	-.824	-.649	***
McKinley	-1.832	.036	-51.42	0	-1.902	-1.762	***
Mora	-.883	.08	-11.10	0	-1.039	-.727	***
Otero	-.791	.029	-27.19	0	-.848	-.734	***
Quay	.312	.043	7.23	0	.228	.397	***
Rio Arriba	-1.143	.032	-36.10	0	-1.206	-1.081	***
Roosevelt	-.069	.041	-1.70	.089	-.148	.01	*
San Juan	-.914	.022	-41.74	0	-.957	-.872	***
San Miguel	-.344	.03	-11.29	0	-.403	-.284	***
Sandoval	-.181	.015	-12.26	0	-.21	-.152	***
Santa Fe	-.3	.012	-24.08	0	-.324	-.275	***
Sierra	-.715	.054	-13.22	0	-.821	-.609	***
Socorro	-.426	.048	-8.90	0	-.52	-.333	***
Taos	-.935	.028	-33.46	0	-.99	-.88	***
Torrance	-.479	.05	-9.50	0	-.578	-.38	***
Union	-.737	.115	-6.40	0	-.962	-.511	***
Valencia	.06	.021	2.84	.005	.018	.101	***
Constant	-2.47	.04	-61.73	0	-2.548	-2.391	***

Mean dependent var	0.173	SD dependent var	0.378
Pseudo r-squared	0.199	Number of obs	937412
Chi-square	126723.936	Prob > chi2	0.000
Akaike crit. (AIC)	691391.712	Bayesian crit. (BIC)	692578.551

**** p<.01, ** p<.05, * p<.1*
