# Supplementary material for: Should I vote-by-mail or in person? The impact of COVID-19 risk factors and partisanship on vote mode decisions in the 2020 presidential election
Source: PLoS One. 2022 Sep 15;17(9):e0274357. doi: 10.1371/journal.pone.0274357 (PMC9477279; doi:10.1371/journal.pone.0274357)
Supplement: S5 Table — (PDF) [file pone.0274357.s005.pdf]

S5 Table. General Election Early Vote Logistic Regression with 2020 as Base Year (Fig 3b)

	Coef.	SE	t-value	p-value	[95% Conf	Interval]	Sig
Age Categories							
30-39 y/o	.153	.026	6.01	0	.103	.203	***
40-49 y/o	.202	.024	8.24	0	.154	.25	***
50-64 y/o	.448	.021	21.00	0	.406	.49	***
65-74 y/o	.813	.023	35.96	0	.769	.858	***
75-84 y/o	.642	.026	24.64	0	.591	.693	***
85+ y/o	.284	.039	7.34	0	.208	.359	***
Political Party							
Independent	-.301	.041	-7.35	0	-.381	-.221	***
Republican	.134	.033	4.04	0	.069	.2	***
Election Year							
2018	-.39	.023	-17.25	0	-.434	-.346	***
2020	-.323	.027	-12.09	0	-.375	-.27	***
Party X Year							
Ind X 2018	-.172	.049	-3.52	0	-.268	-.076	***
Ind X 2020	.404	.054	7.44	0	.298	.511	***
Rep X 2018	-.354	.039	-9.03	0	-.43	-.277	***
Rep X 2020	.798	.044	18.29	0	.713	.884	***
Age X Year							
30-39 X 2018	.019	.029	0.67	.502	-.037	.076	
30-39 X 2020	-.085	.035	-2.47	.014	-.153	-.018	**
40-49 X 2018	.098	.028	3.54	0	.044	.151	***
40-49 X 2020	-.006	.033	-0.20	.845	-.072	.059	
50-64 X 2018	.172	.024	7.11	0	.124	.219	***
50-64 X 2020	-.366	.029	-12.65	0	-.423	-.309	***
65-74 X 2018	.219	.025	8.76	0	.17	.268	***
65-74 X 2020	-1.058	.03	-34.78	0	-1.118	-.998	***
75-84 X 2018	.18	.028	6.51	0	.126	.234	***
75-84 X 2020	-1.219	.035	-34.57	0	-1.288	-1.15	***
85+ X 2018	.096	.038	2.51	.012	.021	.172	**
85+ X 2020	-1.309	.053	-24.76	0	-1.412	-1.205	***
Age X Party							
30-39 X Ind	.038	.053	0.72	.474	-.065	.141	
30-39 X Rep	-.213	.042	-5.05	0	-.296	-.131	***
40-49 X Ind	.168	.052	3.25	.001	.067	.269	***
40-49 X Rep	-.12	.04	-2.97	.003	-.199	-.041	***
50-64 X Ind	.119	.046	2.60	.009	.029	.208	***
50-64 X Rep	-.185	.036	-5.19	0	-.254	-.115	***
65-74 X Ind	-.027	.049	-0.55	.585	-.124	.07	
65-74 X Rep	-.389	.038	-10.32	0	-.463	-.315	***
75-84 X Ind	.044	.063	0.69	.488	-.08	.168	
75-84 X Rep	-.324	.042	-7.68	0	-.407	-.241	***
85+ X Ind	-.091	.109	-0.84	.4	-.304	.121	
85+ X Rep	-.457	.06	-7.60	0	-.575	-.339	***
Party X Age X Year							
Ind X 30-39 X 2018	-.034	.062	-0.54	.587	-.155	.088	
Ind X 30-39 X 2020	-.05	.07	-0.71	.477	-.186	.087	
Ind X 40-49 X 2018	.013	.06	0.21	.831	-.104	.129	
Ind X 40-49 X 2020	-.181	.068	-2.64	.008	-.315	-.047	***
Ind X 50-64 X 2018	.118	.053	2.21	.027	.013	.222	**
Ind X 50-64 X 2020	-.12	.06	-1.98	.047	-.238	-.001	**
Ind X 65-74 X 2018	.154	.056	2.77	.006	.045	.263	***
Ind X 65-74 X 2020	.033	.064	0.51	.61	-.093	.159	

Ind X 75-84 X 2018	.155	.066	2.35	.019	.026	.283	**
Ind X 75-84 X 2020	-.136	.081	-1.67	.096	-.295	.024	*
Ind X 85+ X 2018	.325	.097	3.35	.001	.135	.514	***
Ind X 85+ X 2020	-.02	.139	-0.15	.883	-.292	.251	
Rep X 30-39 X 2018	.046	.049	0.93	.354	-.051	.142	
Rep X 30-39 X 2020	.301	.056	5.38	0	.191	.411	***
Rep X 40-49 X 2018	.132	.047	2.84	.005	.041	.224	***
Rep X 40-49 X 2020	.115	.053	2.16	.031	.011	.219	**
Rep X 50-64 X 2018	.31	.041	7.51	0	.229	.391	***
Rep X 50-64 X 2020	.086	.047	1.85	.064	-.005	.178	*
Rep X 65-74 X 2018	.424	.043	9.88	0	.34	.508	***
Rep X 65-74 X 2020	.361	.049	7.37	0	.265	.457	***
Rep X 75-84 X 2018	.456	.046	9.89	0	.366	.547	***
Rep X 75-84 X 2020	.105	.055	1.93	.054	-.002	.213	*
Rep X 85+ X 2018	.485	.06	8.13	0	.368	.602	***
Rep X 85+ X 2020	-.211	.079	-2.69	.007	-.366	-.057	***
Hispanic	-.234	.006	-38.01	0	-.246	-.222	***
Asian	-.128	.031	-4.09	0	-.19	-.067	***
Black	-.03	.03	-0.98	.326	-.088	.029	
Other Race	-.36	.016	-22.51	0	-.392	-.329	***
Female	-.041	.005	-7.62	0	-.052	-.031	***
Other Sex	.226	.195	1.16	.247	-.157	.608	
County							
Catron	-1.176	.059	-19.87	0	-1.292	-1.06	***
Chaves	.062	.019	3.30	.001	.025	.099	***
Cibola	-.771	.028	-27.26	0	-.826	-.716	***
Colfax	-1.338	.036	-37.42	0	-1.408	-1.268	***
Curry	-.161	.023	-7.03	0	-.206	-.116	***
De Baca	-.789	.077	-10.20	0	-.94	-.637	***
Dona Ana	-.238	.01	-23.46	0	-.258	-.218	***
Eddy	-.147	.018	-7.98	0	-.183	-.111	***
Grant	-.161	.022	-7.32	0	-.204	-.118	***
Guadalupe	-1.024	.06	-17.13	0	-1.141	-.907	***
Harding	-1.169	.109	-10.70	0	-1.384	-.955	***
Hidalgo	-1.207	.066	-18.33	0	-1.336	-1.078	***
Lea	-.146	.02	-7.45	0	-.185	-.108	***
Lincoln	-.455	.026	-17.34	0	-.507	-.404	***
Los Alamos	.014	.022	0.62	.537	-.03	.058	
Luna	-.142	.03	-4.77	0	-.2	-.084	***
McKinley	-.814	.019	-43.65	0	-.85	-.777	***
Mora	-.86	.051	-16.74	0	-.961	-.759	***
Otero	-.248	.019	-13.37	0	-.285	-.212	***
Quay	-1.29	.034	-37.88	0	-1.357	-1.223	***
Rio Arriba	-.785	.021	-37.12	0	-.826	-.743	***
Roosevelt	-.807	.028	-28.47	0	-.863	-.752	***
San Juan	-.241	.013	-19.02	0	-.266	-.216	***
San Miguel	-.865	.025	-35.17	0	-.913	-.816	***
Sandoval	-.087	.011	-8.03	0	-.108	-.066	***
Santa Fe	-.151	.01	-15.70	0	-.17	-.132	***
Sierra	-.322	.036	-8.85	0	-.393	-.25	***
Socorro	-.722	.031	-23.32	0	-.782	-.661	***
Taos	-.277	.02	-13.94	0	-.316	-.238	***
Torrance	-.814	.033	-24.65	0	-.879	-.75	***
Union	-.646	.064	-10.11	0	-.771	-.521	***
Valencia	-.438	.015	-29.11	0	-.468	-.409	***
Constant	.492	.02	24.21	0	.452	.531	***

Mean dependent var	0.578	SD dependent var	0.494
Pseudo r-squared	0.048	Number of obs	937412
Chi-square	47178.149	Prob > chi2	0.000
Akaike crit. (AIC)	1215323.870	Bayesian crit. (BIC)	1216510.708

*** *p<.01*, ** *p<.05*, * *p<.1*
