# Supplementary material for: Should I vote-by-mail or in person? The impact of COVID-19 risk factors and partisanship on vote mode decisions in the 2020 presidential election
Source: PLoS One. 2022 Sep 15;17(9):e0274357. doi: 10.1371/journal.pone.0274357 (PMC9477279; doi:10.1371/journal.pone.0274357)
Supplement: S6 Table — (PDF) [file pone.0274357.s006.pdf]

S6 Table. Logistic Regression Election Day General Election with 2020 as Base Year (Fig 3c)

	Coef.	SE	t-value	p-value	[95% Conf	Interval]	Sig
Age Categories							
30-39 y/o	-.045	.027	-1.63	.103	-.098	.009	
40-49 y/o	-.129	.026	-4.91	0	-.181	-.078	***
50-64 y/o	-.528	.023	-22.86	0	-.573	-.483	***
65-74 y/o	-1.118	.025	-44.95	0	-1.167	-1.069	***
75-84 y/o	-1.152	.029	-39.22	0	-1.21	-1.095	***
85+ y/o	-1.336	.049	-27.54	0	-1.431	-1.241	***
Political Party							
Independent	.361	.043	8.38	0	.277	.446	***
Republican	-.239	.036	-6.65	0	-.31	-.169	***
Election Year							
2018	.319	.024	13.48	0	.273	.365	***
2020	-1.28	.031	-41.73	0	-1.34	-1.22	***
Party X Year							
Ind X 2018	.196	.05	3.95	0	.099	.294	***
Ind X 2020	.165	.059	2.80	.005	.049	.28	***
Rep X 2018	.476	.041	11.70	0	.397	.556	***
Rep X 2020	.345	.05	6.92	0	.247	.442	***
Age X Year							
30-39 X 2018	.02	.03	0.66	.508	-.039	.08	
30-39 X 2020	.002	.039	0.05	.962	-.075	.079	
40-49 X 2018	-.068	.029	-2.33	.02	-.124	-.011	**
40-49 X 2020	-.083	.038	-2.19	.028	-.157	-.009	**
50-64 X 2018	-.16	.025	-6.30	0	-.21	-.11	***
50-64 X 2020	-.087	.033	-2.59	.01	-.152	-.021	***
65-74 X 2018	-.216	.027	-8.11	0	-.268	-.164	***
65-74 X 2020	-.094	.037	-2.55	.011	-.166	-.022	**
75-84 X 2018	-.205	.03	-6.80	0	-.264	-.146	***
75-84 X 2020	-.096	.044	-2.15	.031	-.183	-.009	**
85+ X 2018	-.14	.047	-2.96	.003	-.233	-.047	***
85+ X 2020	-.028	.077	-0.37	.713	-.178	.122	
Age X Party							
30-39 X Ind	-.046	.055	-0.84	.401	-.155	.062	
30-39 X Rep	.213	.045	4.69	0	.124	.302	***
40-49 X Ind	-.177	.055	-3.24	.001	-.284	-.07	***
40-49 X Rep	.149	.043	3.44	.001	.064	.234	***
50-64 X Ind	-.125	.048	-2.59	.01	-.22	-.03	***
50-64 X Rep	.246	.039	6.40	0	.171	.322	***
65-74 X Ind	-.076	.054	-1.41	.158	-.181	.029	
65-74 X Rep	.383	.042	9.21	0	.301	.464	***
75-84 X Ind	-.275	.074	-3.71	0	-.42	-.13	***
75-84 X Rep	.331	.048	6.92	0	.237	.424	***
85+ X Ind	-.078	.141	-0.55	.582	-.355	.199	
85+ X Rep	.381	.076	5.02	0	.232	.529	***
Party X Age X Year							
Ind X 30-39 X 2018	.014	.063	0.22	.826	-.11	.138	
Ind X 30-39 X 2020	-.138	.076	-1.82	.069	-.287	.011	*
Ind X 40-49 X 2018	-.009	.061	-0.14	.888	-.129	.112	
Ind X 40-49 X 2020	-.073	.075	-0.97	.334	-.221	.075	
Ind X 50-64 X 2018	-.102	.055	-1.87	.061	-.209	.005	*
Ind X 50-64 X 2020	-.01	.067	-0.14	.886	-.14	.121	
Ind X 65-74 X 2018	-.117	.059	-1.99	.047	-.232	-.002	**
Ind X 65-74 X 2020	.074	.075	0.98	.326	-.073	.221	

Ind X 75-84 X 2018	-.053	.074	-0.72	.473	-.199	.092	
Ind X 75-84 X 2020	.108	.106	1.02	.308	-.1	.316	
Ind X 85+ X 2018	-.473	.133	-3.57	0	-.733	-.214	***
Ind X 85+ X 2020	-.053	.216	-0.24	.807	-.477	.371	
Rep X 30-39 X 2018	-.101	.051	-1.98	.048	-.202	-.001	**
Rep X 30-39 X 2020	-.292	.063	-4.62	0	-.416	-.168	***
Rep X 40-49 X 2018	-.18	.049	-3.70	0	-.275	-.085	***
Rep X 40-49 X 2020	-.196	.06	-3.23	.001	-.314	-.077	***
Rep X 50-64 X 2018	-.346	.043	-8.02	0	-.43	-.261	***
Rep X 50-64 X 2020	-.093	.054	-1.73	.083	-.198	.012	*
Rep X 65-74 X 2018	-.43	.045	-9.45	0	-.519	-.341	***
Rep X 65-74 X 2020	.033	.058	0.57	.572	-.081	.147	
Rep X 75-84 X 2018	-.463	.05	-9.19	0	-.561	-.364	***
Rep X 75-84 X 2020	-.027	.069	-0.39	.696	-.161	.108	
Rep X 85+ X 2018	-.548	.075	-7.35	0	-.694	-.402	***
Rep X 85+ X 2020	-.393	.121	-3.25	.001	-.629	-.156	***
Hispanic	.44	.008	57.00	0	.425	.455	***
Asian	-.002	.042	-0.05	.964	-.084	.081	
Black	.079	.038	2.07	.039	.004	.154	**
Other Race	.632	.019	33.37	0	.595	.669	***
Female	-.071	.007	-10.25	0	-.084	-.057	***
Other Sex	-.45	.308	-1.46	.145	-1.054	.154	
County							
Catron	1.872	.063	29.71	0	1.748	1.995	***
Chaves	.664	.022	30.46	0	.621	.707	***
Cibola	1.623	.031	52.14	0	1.562	1.684	***
Colfax	2.032	.036	56.65	0	1.962	2.102	***
Curry	1.048	.026	40.22	0	.997	1.099	***
De Baca	1.728	.085	20.27	0	1.561	1.895	***
Dona Ana	.677	.013	52.74	0	.651	.702	***
Eddy	.881	.021	41.12	0	.839	.924	***
Grant	.732	.027	27.49	0	.68	.784	***
Guadalupe	1.226	.062	19.65	0	1.104	1.348	***
Harding	1.302	.128	10.18	0	1.051	1.552	***
Hidalgo	1.898	.067	28.50	0	1.768	2.029	***
Lea	.861	.023	37.88	0	.817	.906	***
Lincoln	1.08	.031	35.41	0	1.021	1.14	***
Los Alamos	.341	.03	11.49	0	.283	.4	***
Luna	.785	.036	21.97	0	.715	.855	***
McKinley	1.925	.021	90.66	0	1.884	1.967	***
Mora	1.666	.055	30.15	0	1.558	1.774	***
Otero	.943	.022	42.78	0	.9	.986	***
Quay	1.488	.05	29.91	0	1.39	1.585	***
Rio Arriba	1.686	.023	72.67	0	1.641	1.732	***
Roosevelt	1.21	.037	32.47	0	1.137	1.283	***
San Juan	.958	.015	63.18	0	.928	.988	***
San Miguel	1.404	.026	53.20	0	1.352	1.455	***
Sandoval	.314	.015	21.50	0	.285	.342	***
Santa Fe	.489	.013	37.68	0	.463	.514	***
Sierra	1.019	.044	23.40	0	.934	1.104	***
Socorro	1.302	.036	36.64	0	1.232	1.372	***
Taos	1.065	.024	44.23	0	1.018	1.113	***
Torrance	1.455	.037	39.36	0	1.382	1.527	***
Union	1.386	.071	19.43	0	1.247	1.526	***
Valencia	.633	.019	33.97	0	.597	.67	***
Constant	-1.083	.022	-48.53	0	-1.127	-1.039	***

Mean dependent var	0.249	SD dependent var	0.432
Pseudo r-squared	0.131	Number of obs	937412
Chi-square	100403.762	Prob > chi2	0.000
Akaike crit. (AIC)	914989.416	Bayesian crit. (BIC)	916176.255

*** *p<.01*, ** *p<.05*, * *p<.1*
