# Supplementary material for: Should I vote-by-mail or in person? The impact of COVID-19 risk factors and partisanship on vote mode decisions in the 2020 presidential election
Source: PLoS One. 2022 Sep 15;17(9):e0274357. doi: 10.1371/journal.pone.0274357 (PMC9477279; doi:10.1371/journal.pone.0274357)
Supplement: S7 Table — (PDF) [file pone.0274357.s007.pdf]

S7 Table. Multinomial Logistic Regression Vote Mode General Election 2016 (Fig 3g)

	Coef.	SE	t-value	p-value	[95% Conf	Interval]	Sig
VBM							
Age Categories							
30-39 y/o	.551	.033	-9.82	0	.489	.621	***
40-49 y/o	.494	.029	-11.96	0	.44	.554	***
50-64 y/o	.939	.043	-1.38	.168	.859	1.027	
65-74 y/o	2.195	.101	17.02	0	2.005	2.403	***
75-84 y/o	3.287	.166	23.62	0	2.978	3.628	***
85+ y/o	7.444	.489	30.53	0	6.544	8.467	***
Political Party							
Independent	.677	.062	-4.28	0	.566	.809	***
Republican	1.08	.074	1.12	.264	.944	1.235	
Age X Party							
30-39 X Ind	1.106	.145	0.77	.441	.856	1.43	
30-39 X Rep	.875	.086	-1.36	.175	.722	1.061	
40-49 X Ind	1.643	.206	3.96	0	1.285	2.1	***
40-49 X Rep	1.155	.107	1.56	.12	.963	1.385	
50-64 X Ind	1.685	.171	5.16	0	1.382	2.055	***
50-64 X Rep	1.216	.09	2.64	.008	1.052	1.406	***
65-74 X Ind	1.823	.188	5.83	0	1.49	2.231	***
65-74 X Rep	1.175	.089	2.13	.033	1.013	1.363	**
75-84 X Ind	2.188	.262	6.53	0	1.729	2.767	***
75-84 X Rep	1.12	.091	1.40	.163	.955	1.313	
85+ X Ind	1.675	.297	2.91	.004	1.184	2.371	***
85+ X Rep	1.072	.11	0.68	.499	.876	1.312	
Hispanic	.648	.011	-24.66	0	.626	.671	***
Asian	1.358	.115	3.62	0	1.15	1.603	***
Black	.888	.077	-1.37	.171	.75	1.053	
Other Race	.294	.016	-22.99	0	.265	.326	***
Female	1.316	.02	17.82	0	1.277	1.356	***
Other Sex	.487	.527	-0.66	.506	.059	4.058	
Constant	.185	.008	-39.04	0	.17	.202	***
Early Vote							
Age Categories							
30-39 y/o	1.069	.029	2.50	.012	1.015	1.127	**
40-49 y/o	1.083	.028	3.10	.002	1.03	1.139	***
50-64 y/o	1.429	.032	15.89	0	1.367	1.493	***
65-74 y/o	2.424	.059	36.54	0	2.311	2.542	***
75-84 y/o	2.309	.067	29.07	0	2.183	2.444	***
85+ y/o	2.43	.118	18.33	0	2.21	2.672	***
Political Party							
Independent	.707	.03	-8.14	0	.65	.768	***
Republican	1.096	.038	2.62	.009	1.023	1.174	***
Age X Party							
30-39 X Ind	1.084	.059	1.47	.141	.974	1.206	
30-39 X Rep	.795	.035	-5.17	0	.729	.867	***
40-49 X Ind	1.295	.07	4.80	0	1.165	1.44	***
40-49 X Rep	.924	.039	-1.85	.064	.85	1.005	*
50-64 X Ind	1.258	.06	4.79	0	1.145	1.381	***
50-64 X Rep	.917	.035	-2.30	.022	.852	.987	**
65-74 X Ind	1.127	.06	2.26	.024	1.016	1.251	**
65-74 X Rep	.766	.031	-6.55	0	.707	.829	***
75-84 X Ind	1.361	.1	4.17	0	1.178	1.573	***
75-84 X Rep	.839	.04	-3.73	0	.765	.92	***

85+ X Ind	1.114	.159	0.75	.451	.842	1.475	
85+ X Rep	.765	.058	-3.50	0	.659	.889	***
Hispanic	.642	.006	-49.08	0	.631	.653	***
Asian	1.026	.051	0.52	.602	.931	1.132	
Black	.908	.043	-2.05	.04	.828	.996	**
Other Race	.377	.008	-45.33	0	.361	.393	***
Female	1.068	.009	8.01	0	1.051	1.086	***
Other Sex	1.462	.64	0.87	.385	.62	3.446	
Constant	1.737	.038	25.38	0	1.665	1.813	***

Mean dependent var	2.217	SD dependent var	0.563
Pseudo r-squared	0.034	Number of obs	312472
Chi-square	17637.650	Prob > chi2	0.000
Akaike crit. (AIC)	506231.039	Bayesian crit. (BIC)	506806.261

**** p<.01, ** p<.05, * p<.1*
