# Supplementary material for: Should I vote-by-mail or in person? The impact of COVID-19 risk factors and partisanship on vote mode decisions in the 2020 presidential election
Source: PLoS One. 2022 Sep 15;17(9):e0274357. doi: 10.1371/journal.pone.0274357 (PMC9477279; doi:10.1371/journal.pone.0274357)
Supplement: S8 Table — (PDF) [file pone.0274357.s008.pdf]

S8 Table. Multinomial Logistic Regression Vote Mode General Election 2018 (Fig 3h)

	Coef.	SE	t-value	p-value	[95% Conf	Interval]	Sig
VBM							
Age Categories							
30-39 y/o	.566	.03	-10.91	0	.511	.627	***
40-49 y/o	.559	.028	-11.60	0	.507	.617	***
50-64 y/o	1.028	.041	0.70	.482	.952	1.111	
65-74 y/o	2.269	.092	20.21	0	2.096	2.457	***
75-84 y/o	3.343	.149	27.07	0	3.064	3.649	***
85+ y/o	6.955	.413	32.70	0	6.192	7.812	***
Political Party							
Independent	.591	.048	-6.49	0	.504	.693	***
Republican	.636	.041	-7.04	0	.56	.721	***
Age X Party							
30-39 X Ind	1.192	.136	1.54	.122	.954	1.491	
30-39 X Rep	.912	.084	-1.00	.316	.761	1.092	
40-49 X Ind	1.509	.167	3.71	0	1.214	1.874	***
40-49 X Rep	1.181	.102	1.94	.053	.998	1.398	*
50-64 X Ind	1.64	.148	5.46	0	1.373	1.958	***
50-64 X Rep	1.495	.104	5.80	0	1.305	1.712	***
65-74 X Ind	1.82	.169	6.44	0	1.517	2.184	***
65-74 X Rep	1.583	.113	6.45	0	1.377	1.821	***
75-84 X Ind	2.046	.223	6.57	0	1.653	2.533	***
75-84 X Rep	1.652	.126	6.57	0	1.422	1.918	***
85+ X Ind	2.445	.417	5.24	0	1.749	3.416	***
85+ X Rep	1.841	.178	6.30	0	1.523	2.226	***
Hispanic	.802	.013	-14.02	0	.778	.827	***
Asian	1.464	.118	4.74	0	1.25	1.714	***
Black	.979	.078	-0.27	.789	.838	1.144	
Other Race	.339	.017	-22.10	0	.308	.373	***
Female	1.24	.018	15.03	0	1.205	1.275	***
Other Sex	3.63	3.031	1.54	.123	.707	18.648	
Constant	.197	.007	-43.04	0	.183	.212	***
Early Vote							
Age Categories							
30-39 y/o	1.081	.029	2.94	.003	1.026	1.139	***
40-49 y/o	1.194	.031	6.93	0	1.136	1.256	***
50-64 y/o	1.712	.038	24.01	0	1.639	1.789	***
65-74 y/o	3.045	.073	46.23	0	2.905	3.192	***
75-84 y/o	2.823	.08	36.41	0	2.67	2.985	***
85+ y/o	2.665	.125	20.82	0	2.43	2.922	***
Political Party							
Independent	.572	.025	-12.67	0	.525	.624	***
Republican	.693	.024	-10.61	0	.648	.742	***
Age X Party							
30-39 X Ind	1.055	.059	0.97	.334	.946	1.178	
30-39 X Rep	.866	.038	-3.27	.001	.795	.944	***
40-49 X Ind	1.296	.071	4.74	0	1.164	1.443	***
40-49 X Rep	1.089	.045	2.04	.042	1.003	1.181	**
50-64 X Ind	1.384	.068	6.64	0	1.257	1.523	***
50-64 X Rep	1.272	.047	6.51	0	1.183	1.367	***
65-74 X Ind	1.261	.068	4.32	0	1.135	1.4	***
65-74 X Rep	1.165	.046	3.83	0	1.077	1.26	***
75-84 X Ind	1.436	.103	5.03	0	1.247	1.654	***
75-84 X Rep	1.324	.061	6.08	0	1.209	1.449	***

85+ X Ind	1.765	.261	3.85	0	1.322	2.357	***
85+ X Rep	1.318	.098	3.71	0	1.139	1.525	***
Hispanic	.6	.005	-57.43	0	.589	.61	***
Asian	1.169	.057	3.18	.001	1.062	1.287	***
Black	.898	.041	-2.36	.018	.821	.982	**
Other Race	.399	.009	-42.12	0	.382	.416	***
Female	.98	.008	-2.51	.012	.965	.996	**
Other Sex	3.813	2.407	2.12	.034	1.106	13.138	**
Constant	1.308	.028	12.36	0	1.253	1.364	***

Mean dependent var	2.256	SD dependent var	0.601
Pseudo r-squared	0.045	Number of obs	312471
Chi-square	25001.885	Prob > chi2	0.000
Akaike crit. (AIC)	535378.626	Bayesian crit. (BIC)	535953.848

*** *p<.01*, ** *p<.05*, * *p<.1*
