# Supplementary material for: Should I vote-by-mail or in person? The impact of COVID-19 risk factors and partisanship on vote mode decisions in the 2020 presidential election
Source: PLoS One. 2022 Sep 15;17(9):e0274357. doi: 10.1371/journal.pone.0274357 (PMC9477279; doi:10.1371/journal.pone.0274357)
Supplement: S9 Table — (PDF) [file pone.0274357.s009.pdf]

S9 Table. Multinomial Logistic Regression Vote Mode General Election 2020 (Fig 3i)

	Coef.	SE	t-value	p-value	[95% Conf	Interval]	Sig
VBM							
Age Categories							
30-39 y/o	.958	.036	-1.14	.256	.889	1.032	
40-49 y/o	.991	.037	-0.25	.806	.922	1.065	
50-64 y/o	1.567	.05	13.97	0	1.471	1.668	***
65-74 y/o	3.425	.123	34.27	0	3.192	3.675	***
75-84 y/o	4.015	.176	31.73	0	3.685	4.375	***
85+ y/o	5.28	.401	21.90	0	4.55	6.128	***
Political Party							
Independent	.474	.028	-12.65	0	.422	.532	***
Republican	.273	.015	-22.98	0	.245	.305	***
Age X Party							
30-39 X Ind	1.316	.101	3.58	0	1.132	1.53	***
30-39 X Rep	.928	.068	-1.02	.308	.804	1.071	
40-49 X Ind	1.525	.117	5.49	0	1.312	1.773	***
40-49 X Rep	1.142	.079	1.90	.057	.996	1.308	*
50-64 X Ind	1.385	.093	4.82	0	1.213	1.581	***
50-64 X Rep	1.318	.08	4.56	0	1.17	1.483	***
65-74 X Ind	1.219	.093	2.59	.01	1.049	1.417	***
65-74 X Rep	1.14	.074	2.02	.043	1.004	1.296	**
75-84 X Ind	1.541	.167	3.98	0	1.245	1.906	***
75-84 X Rep	1.656	.124	6.75	0	1.43	1.917	***
85+ X Ind	1.556	.339	2.03	.043	1.015	2.386	**
85+ X Rep	3.057	.379	9.01	0	2.398	3.899	***
Hispanic	.525	.007	-47.04	0	.511	.539	***
Asian	1.661	.14	6.03	0	1.409	1.959	***
Black	.857	.064	-2.07	.039	.741	.992	**
Other Race	.255	.008	-43.70	0	.24	.272	***
Female	1.255	.016	18.05	0	1.224	1.286	***
Other Sex	1.532	.608	1.07	.283	.703	3.335	
Constant	2.914	.091	34.35	0	2.742	3.098	***
Early Vote							
Age Categories							
30-39 y/o	1.034	.038	0.92	.359	.963	1.111	
40-49 y/o	1.16	.041	4.19	0	1.082	1.243	***
50-64 y/o	1.423	.044	11.34	0	1.339	1.513	***
65-74 y/o	2.071	.073	20.62	0	1.933	2.219	***
75-84 y/o	1.679	.074	11.74	0	1.54	1.831	***
85+ y/o	1.431	.115	4.48	0	1.223	1.674	***
Political Party							
Independent	.716	.038	-6.27	0	.645	.795	***
Republican	1.213	.055	4.29	0	1.111	1.325	***
Age X Party							
30-39 X Ind	1.19	.083	2.49	.013	1.038	1.364	**
30-39 X Rep	1.067	.062	1.12	.264	.953	1.194	
40-49 X Ind	1.345	.094	4.24	0	1.173	1.542	***
40-49 X Rep	1.094	.061	1.61	.107	.981	1.22	
50-64 X Ind	1.262	.078	3.76	0	1.118	1.424	***
50-64 X Rep	1.006	.049	0.12	.906	.914	1.107	
65-74 X Ind	1.066	.077	0.88	.379	.925	1.228	
65-74 X Rep	.819	.045	-3.65	0	.735	.912	***
75-84 X Ind	1.221	.132	1.84	.066	.987	1.51	*
75-84 X Rep	.94	.063	-0.92	.356	.825	1.072	

85+ X Ind	1.203	.276	0.80	.421	.767	1.886	
85+ X Rep	1.025	.127	0.20	.844	.803	1.308	
Hispanic	.608	.008	-38.56	0	.593	.624	***
Asian	1.149	.096	1.67	.095	.976	1.353	*
Black	.962	.068	-0.56	.578	.837	1.104	
Other Race	.427	.012	-31.49	0	.405	.45	***
Female	1.117	.013	9.37	0	1.092	1.143	***
Other Sex	1.51	.583	1.07	.286	.708	3.218	
Constant	3.604	.108	42.77	0	3.398	3.822	***

Mean dependent var	1.756	SD dependent var	0.645
Pseudo r-squared	0.062	Number of obs	312469
Chi-square	36975.938	Prob > chi2	0.000
Akaike crit. (AIC)	560177.776	Bayesian crit. (BIC)	560752.998

*** *p<.01*, ** *p<.05*, * *p<.1*
