# Supplementary material for: Should I vote-by-mail or in person? The impact of COVID-19 risk factors and partisanship on vote mode decisions in the 2020 presidential election
Source: PLoS One. 2022 Sep 15;17(9):e0274357. doi: 10.1371/journal.pone.0274357 (PMC9477279; doi:10.1371/journal.pone.0274357)
Supplement: S10 Table — (PDF) [file pone.0274357.s010.pdf]

S10 Table. Logistic Regression Vote by Mail Primary Election with 2020 as Base Year (Fig 5a)

	Coef.	SE	t-value	p-value	[95% Conf	Interval]	Sig
Age Categories							
30-39 y/o	.238	.061	3.89	0	.118	.358	***
40-49 y/o	.356	.058	6.16	0	.243	.469	***
50-64 y/o	.02	.051	0.40	.692	-.08	.121	
65-74 y/o	-.488	.052	-9.48	0	-.589	-.387	***
75-84 y/o	-.556	.054	-10.27	0	-.663	-.45	***
85+ y/o	-.696	.07	-9.99	0	-.833	-.56	***
Political Party							
Democrat	.563	.119	4.73	0	.33	.797	***
Election Year							
2018	-3.395	.139	-24.35	0	-3.668	-3.122	***
2020	.498	.076	6.51	0	.348	.648	***
Party X Year							
Dem X 2018	-.202	.292	-0.69	.491	-.775	.371	
Dem X 2020	-2.104	.182	-11.57	0	-2.461	-1.748	***
Age X Year							
30-39 X 2018	-.637	.183	-3.48	0	-.995	-.279	***
30-39 X 2020	-.145	.095	-1.53	.127	-.331	.041	
40-49 X 2018	-1.044	.177	-5.89	0	-1.391	-.696	***
40-49 X 2020	-.365	.089	-4.11	0	-.54	-.191	***
50-64 X 2018	-.307	.146	-2.11	.035	-.593	-.021	**
50-64 X 2020	.183	.079	2.30	.021	.027	.338	**
65-74 X 2018	.631	.145	4.36	0	.347	.914	***
65-74 X 2020	1.261	.08	15.72	0	1.104	1.418	***
75-84 X 2018	1.248	.148	8.43	0	.957	1.538	***
75-84 X 2020	1.389	.085	16.28	0	1.222	1.556	***
85+ X 2018	2.376	.165	14.39	0	2.052	2.699	***
85+ X 2020	1.917	.117	16.34	0	1.687	2.147	***
Age X Party							
30-39 X Dem	.015	.147	0.10	.919	-.273	.303	
40-49 X Dem	-.267	.134	-2.00	.046	-.529	-.005	**
50-64 X Dem	-.395	.121	-3.26	.001	-.633	-.158	***
65-74 X Dem	-.432	.122	-3.54	0	-.671	-.193	***
75-84 X Dem	-.481	.125	-3.86	0	-.725	-.236	***
85+ X Dem	-.469	.145	-3.23	.001	-.754	-.185	***
Party X Age X Year							
Dem X 30-39 X 2018	-.452	.402	-1.12	.261	-1.24	.337	
Dem X 30-39 X 2020	-.506	.227	-2.23	.026	-.951	-.061	**
Dem X 40-49 X 2018	.196	.354	0.55	.58	-.498	.89	
Dem X 40-49 X 2020	.085	.204	0.42	.678	-.315	.485	
Dem X 50-64 X 2018	.423	.301	1.41	.159	-.166	1.013	
Dem X 50-64 X 2020	.4	.186	2.15	.031	.036	.765	**
Dem X 65-74 X 2018	.424	.3	1.41	.157	-.164	1.012	
Dem X 65-74 X 2020	.353	.187	1.89	.059	-.013	.718	*
Dem X 75-84 X 2018	.246	.304	0.81	.418	-.349	.842	
Dem X 75-84 X 2020	.84	.191	4.40	0	.466	1.215	***
Dem X 85+ X 2018	.315	.328	0.96	.338	-.328	.958	
Dem X 85+ X 2020	1.181	.229	5.15	0	.731	1.63	***
Hispanic	-.083	.011	-7.82	0	-.104	-.062	***
Asian	0	.065	0.00	.999	-.128	.128	
Black	.043	.05	0.86	.388	-.054	.14	
Other Race	-.068	.029	-2.32	.02	-.125	-.01	**
Female	.065	.009	7.15	0	.047	.083	***

Other Sex	.12	.462	0.26	.795	-.785	1.025	
County							
Catron	.043	.078	0.55	.583	-.109	.195	
Chaves	-.245	.032	-7.72	0	-.308	-.183	***
Cibola	.065	.044	1.50	.133	-.02	.151	
Colfax	.54	.049	11.12	0	.445	.636	***
Curry	-.062	.04	-1.55	.122	-.141	.017	
De Baca	.271	.102	2.64	.008	.07	.471	***
Dona Ana	-.294	.02	-14.97	0	-.332	-.256	***
Eddy	-.163	.034	-4.77	0	-.23	-.096	***
Grant	-.174	.031	-5.55	0	-.236	-.113	***
Guadalupe	.358	.081	4.40	0	.199	.517	***
Harding	.727	.133	5.45	0	.466	.988	***
Hidalgo	.299	.111	2.69	.007	.081	.518	***
Lea	-.099	.035	-2.86	.004	-.168	-.031	***
Lincoln	.236	.044	5.42	0	.151	.321	***
Los Alamos	.033	.036	0.93	.351	-.037	.104	
Luna	-.225	.047	-4.75	0	-.317	-.132	***
McKinley	.048	.031	1.56	.12	-.012	.109	
Mora	-.134	.059	-2.25	.025	-.25	-.017	**
Otero	-.067	.031	-2.17	.03	-.128	-.007	**
Quay	-.11	.058	-1.91	.056	-.223	.003	*
Rio Arriba	.018	.027	0.67	.503	-.035	.071	
Roosevelt	.11	.048	2.31	.021	.017	.203	**
San Juan	.204	.023	8.90	0	.159	.249	***
San Miguel	.312	.033	9.59	0	.249	.376	***
Sandoval	-.109	.019	-5.86	0	-.146	-.073	***
Santa Fe	.141	.015	9.34	0	.111	.17	***
Sierra	.042	.054	0.78	.438	-.064	.149	
Socorro	.017	.044	0.39	.694	-.069	.104	
Taos	-.069	.028	-2.45	.014	-.124	-.014	**
Torrance	.245	.05	4.95	0	.148	.342	***
Union	-.064	.096	-0.66	.507	-.252	.125	
Valencia	.247	.027	9.27	0	.195	.299	***
Constant	.182	.05	3.62	0	.083	.28	***

Mean dependent var	0.406	SD dependent var	0.491
Pseudo r-squared	0.289	Number of obs	269369
Chi-square	60963.428	Prob > chi2	0.000
Akaike crit. (AIC)	258639.146	Bayesian crit. (BIC)	259479.453

*** *p<.01*, ** *p<.05*, * *p<.1*
