# Supplementary material for: Should I vote-by-mail or in person? The impact of COVID-19 risk factors and partisanship on vote mode decisions in the 2020 presidential election
Source: PLoS One. 2022 Sep 15;17(9):e0274357. doi: 10.1371/journal.pone.0274357 (PMC9477279; doi:10.1371/journal.pone.0274357)
Supplement: S11 Table — (PDF) [file pone.0274357.s011.pdf]

S11 Table. Logistic Regression Early Vote Primary Election with 2020 as Base Year (Fig 5b)

	Coef.	SE	t-value	p-value	[95% Conf	Interval]	Sig
Age Categories							
30-39 y/o	-.176	.062	-2.85	.004	-.297	-.055	***
40-49 y/o	-.241	.058	-4.14	0	-.355	-.127	***
50-64 y/o	.073	.052	1.41	.159	-.029	.174	
65-74 y/o	.492	.052	9.49	0	.391	.594	***
75-84 y/o	.462	.054	8.48	0	.355	.569	***
85+ y/o	.234	.069	3.37	.001	.098	.37	***
Political Party							
Democrat	-.658	.126	-5.21	0	-.906	-.411	***
Election Year							
2018	-.33	.062	-5.33	0	-.451	-.209	***
2020	-1.925	.097	-19.82	0	-2.116	-1.735	***
Party X Year							
Dem X 2018	.812	.141	5.77	0	.536	1.089	***
Dem X 2020	2.226	.18	12.34	0	1.873	2.58	***
Age X Year							
30-39 X 2018	.14	.075	1.85	.064	-.008	.287	*
30-39 X 2020	-.13	.123	-1.05	.293	-.371	.112	
40-49 X 2018	.347	.071	4.87	0	.207	.486	***
40-49 X 2020	.517	.111	4.67	0	.3	.734	***
50-64 X 2018	.387	.064	6.08	0	.262	.512	***
50-64 X 2020	.41	.1	4.11	0	.214	.606	***
65-74 X 2018	.366	.064	5.75	0	.241	.491	***
65-74 X 2020	-.214	.1	-2.13	.033	-.411	-.017	**
75-84 X 2018	.349	.066	5.30	0	.22	.478	***
75-84 X 2020	-.246	.105	-2.35	.019	-.452	-.041	**
85+ X 2018	.228	.08	2.86	.004	.072	.384	***
85+ X 2020	-.401	.137	-2.93	.003	-.668	-.133	***
Age X Party							
30-39 X Dem	.118	.155	0.76	.447	-.185	.421	
40-49 X Dem	.345	.141	2.45	.014	.069	.621	**
50-64 X Dem	.395	.129	3.07	.002	.143	.647	***
65-74 X Dem	.437	.129	3.39	.001	.184	.69	***
75-84 X Dem	.452	.131	3.44	.001	.195	.71	***
85+ X Dem	.362	.151	2.40	.017	.066	.659	**
Party X Age X Year							
Dem X 30-39 X 2018	-.316	.175	-1.80	.072	-.66	.028	*
Dem X 30-39 X 2020	.211	.221	0.95	.34	-.222	.643	
Dem X 40-49 X 2018	-.616	.158	-3.90	0	-.925	-.306	***
Dem X 40-49 X 2020	-.543	.2	-2.71	.007	-.936	-.151	***
Dem X 50-64 X 2018	-.777	.144	-5.41	0	-1.058	-.496	***
Dem X 50-64 X 2020	-.676	.184	-3.68	0	-1.036	-.315	***
Dem X 65-74 X 2018	-.817	.144	-5.68	0	-1.099	-.535	***
Dem X 65-74 X 2020	-.588	.185	-3.19	.001	-.95	-.226	***
Dem X 75-84 X 2018	-.816	.146	-5.59	0	-1.102	-.53	***
Dem X 75-84 X 2020	-.878	.189	-4.64	0	-1.248	-.507	***
Dem X 85+ X 2018	-.679	.164	-4.13	0	-1.001	-.357	***
Dem X 85+ X 2020	-1.148	.229	-5.01	0	-1.597	-.699	***
Hispanic	-.111	.012	-9.09	0	-.135	-.087	***
Asian	-.01	.075	-0.14	.889	-.157	.136	
Black	-.037	.058	-0.63	.53	-.151	.078	
Other Race	-.172	.036	-4.83	0	-.242	-.102	***
Female	-.025	.01	-2.35	.019	-.045	-.004	**

Other Sex	.541	.418	1.29	.196	-.279	1.361	
County							
Catron	-.717	.099	-7.24	0	-.911	-.523	***
Chaves	-.035	.035	-1.02	.309	-.104	.033	
Cibola	-.645	.057	-11.32	0	-.757	-.534	***
Colfax	-1.507	.074	-20.45	0	-1.651	-1.362	***
Curry	-.394	.049	-8.03	0	-.49	-.298	***
De Baca	-1.262	.164	-7.72	0	-1.582	-.941	***
Dona Ana	-.151	.023	-6.71	0	-.195	-.107	***
Eddy	-.365	.04	-9.14	0	-.443	-.287	***
Grant	-.187	.036	-5.24	0	-.257	-.117	***
Guadalupe	-.559	.093	-5.99	0	-.742	-.376	***
Harding	-.866	.161	-5.39	0	-1.181	-.551	***
Hidalgo	-.224	.121	-1.86	.063	-.46	.012	*
Lea	-.3	.039	-7.62	0	-.377	-.223	***
Lincoln	-.763	.054	-14.23	0	-.868	-.658	***
Los Alamos	-.021	.039	-0.55	.581	-.097	.055	
Luna	-.127	.054	-2.33	.02	-.234	-.02	**
McKinley	-.847	.04	-21.02	0	-.926	-.768	***
Mora	-.363	.072	-5.06	0	-.504	-.223	***
Otero	-.252	.036	-6.98	0	-.323	-.181	***
Quay	-1.127	.068	-16.63	0	-1.26	-.995	***
Rio Arriba	-.578	.034	-16.85	0	-.645	-.51	***
Roosevelt	-.887	.062	-14.27	0	-1.009	-.766	***
San Juan	-.534	.027	-19.61	0	-.587	-.48	***
San Miguel	-.803	.041	-19.44	0	-.884	-.722	***
Sandoval	-.106	.021	-5.10	0	-.147	-.065	***
Santa Fe	-.422	.017	-24.32	0	-.456	-.388	***
Sierra	-.241	.062	-3.91	0	-.362	-.12	***
Socorro	-.538	.055	-9.85	0	-.645	-.431	***
Taos	-.315	.033	-9.55	0	-.38	-.251	***
Torrance	-.762	.063	-12.02	0	-.887	-.638	***
Union	-.357	.112	-3.20	.001	-.575	-.138	***
Valencia	-.556	.032	-17.59	0	-.617	-.494	***
Constant	-.107	.051	-2.10	.036	-.206	-.007	**

Mean dependent var	0.352	SD dependent var	0.478
Pseudo r-squared	0.093	Number of obs	269369
Chi-square	21877.290	Prob > chi2	0.000
Akaike crit. (AIC)	317022.200	Bayesian crit. (BIC)	317862.507

**** p<.01, ** p<.05, * p<.1*
