# Supplementary material for: Should I vote-by-mail or in person? The impact of COVID-19 risk factors and partisanship on vote mode decisions in the 2020 presidential election
Source: PLoS One. 2022 Sep 15;17(9):e0274357. doi: 10.1371/journal.pone.0274357 (PMC9477279; doi:10.1371/journal.pone.0274357)
Supplement: S12 Table — (PDF) [file pone.0274357.s012.pdf]

S12 Table. Logistic Regression Election Day Primary with 2020 as Base Year (Fig 5c)

	Coef.	SE	t-value	p-value	[95% Conf	Interval]	Sig
Age Categories							
30-39 y/o	.235	.063	3.72	0	.111	.359	***
40-49 y/o	.281	.06	4.70	0	.164	.399	***
50-64 y/o	-.102	.053	-1.91	.056	-.206	.002	*
65-74 y/o	-.597	.053	-11.16	0	-.702	-.492	***
75-84 y/o	-.713	.056	-12.69	0	-.823	-.603	***
85+ y/o	-.838	.072	-11.64	0	-.979	-.697	***
Political Party							
Democrat	.401	.121	3.31	.001	.163	.639	***
Election Year							
2018	.349	.062	5.63	0	.227	.471	***
2020	-1.412	.073	-19.44	0	-1.554	-1.27	***
Party X Year							
Dem X 2018	-.751	.135	-5.58	0	-1.015	-.487	***
Dem X 2020	.13	.156	0.83	.406	-.176	.436	
Age X Year							
30-39 X 2018	-.151	.076	-2.00	.046	-.3	-.003	**
30-39 X 2020	-.251	.089	-2.82	.005	-.425	-.077	***
40-49 X 2018	-.345	.071	-4.83	0	-.484	-.205	***
40-49 X 2020	-.508	.084	-6.02	0	-.674	-.343	***
50-64 X 2018	-.383	.064	-6.00	0	-.508	-.258	***
50-64 X 2020	-.616	.076	-8.15	0	-.764	-.468	***
65-74 X 2018	-.354	.064	-5.53	0	-.479	-.228	***
65-74 X 2020	-.942	.078	-12.07	0	-1.095	-.789	***
75-84 X 2018	-.34	.066	-5.13	0	-.47	-.21	***
75-84 X 2020	-.868	.085	-10.21	0	-1.035	-.701	***
85+ X 2018	-.294	.081	-3.65	0	-.452	-.136	***
85+ X 2020	-1.06	.133	-7.98	0	-1.32	-.8	***
Age X Party							
30-39 X Dem	-.092	.149	-0.62	.537	-.385	.2	
40-49 X Dem	-.261	.136	-1.92	.055	-.527	.006	*
50-64 X Dem	-.262	.124	-2.12	.034	-.504	-.019	**
65-74 X Dem	-.303	.124	-2.44	.015	-.547	-.06	**
75-84 X Dem	-.273	.127	-2.15	.032	-.522	-.024	**
85+ X Dem	-.232	.148	-1.57	.117	-.521	.058	
Party X Age X Year							
Dem X 30-39 X 2018	.317	.168	1.89	.059	-.012	.645	*
Dem X 30-39 X 2020	.369	.189	1.96	.05	-.001	.739	*
Dem X 40-49 X 2018	.581	.151	3.84	0	.284	.878	***
Dem X 40-49 X 2020	.563	.174	3.23	.001	.222	.905	***
Dem X 50-64 X 2018	.74	.137	5.39	0	.471	1.01	***
Dem X 50-64 X 2020	.589	.16	3.69	0	.276	.902	***
Dem X 65-74 X 2018	.777	.138	5.64	0	.507	1.047	***
Dem X 65-74 X 2020	.899	.162	5.55	0	.582	1.216	***
Dem X 75-84 X 2018	.804	.14	5.74	0	.529	1.079	***
Dem X 75-84 X 2020	.529	.169	3.14	.002	.199	.86	***
Dem X 85+ X 2018	.626	.16	3.92	0	.313	.939	***
Dem X 85+ X 2020	.422	.226	1.86	.062	-.022	.865	*
Hispanic	.272	.013	21.22	0	.247	.297	***
Asian	-.018	.083	-0.22	.828	-.182	.145	
Black	.053	.063	0.83	.406	-.071	.177	
Other Race	.37	.035	10.48	0	.301	.439	***
Female	-.081	.011	-7.24	0	-.102	-.059	***

Other Sex	-.666	.474	-1.40	.16	-1.595	.264	
County							
Catron	1.269	.096	13.26	0	1.082	1.457	***
Chaves	.701	.036	19.39	0	.63	.772	***
Cibola	1.095	.055	19.80	0	.986	1.203	***
Colfax	1.698	.056	30.10	0	1.588	1.809	***
Curry	1.085	.051	21.37	0	.985	1.184	***
De Baca	1.727	.143	12.03	0	1.446	2.008	***
Dona Ana	.758	.024	31.36	0	.71	.805	***
Eddy	1.066	.041	26.25	0	.986	1.146	***
Grant	.681	.038	18.14	0	.608	.755	***
Guadalupe	.667	.089	7.52	0	.494	.841	***
Harding	.721	.15	4.79	0	.426	1.015	***
Hidalgo	.592	.096	6.18	0	.404	.779	***
Lea	.91	.041	22.34	0	.83	.99	***
Lincoln	1.124	.051	22.22	0	1.025	1.223	***
Los Alamos	.204	.045	4.58	0	.117	.291	***
Luna	.69	.058	11.93	0	.577	.804	***
McKinley	1.448	.037	38.83	0	1.374	1.521	***
Mora	.842	.073	11.60	0	.699	.984	***
Otero	.757	.038	20.14	0	.683	.831	***
Quay	.945	.082	11.52	0	.784	1.106	***
Rio Arriba	1.116	.034	32.87	0	1.049	1.182	***
Roosevelt	1.351	.065	20.70	0	1.223	1.479	***
San Juan	.801	.027	29.19	0	.747	.855	***
San Miguel	1.061	.038	28.22	0	.988	1.135	***
Sandoval	.324	.024	13.61	0	.278	.371	***
Santa Fe	.717	.018	39.49	0	.682	.753	***
Sierra	.672	.066	10.24	0	.543	.8	***
Socorro	.917	.054	16.87	0	.811	1.024	***
Taos	.89	.033	26.64	0	.825	.955	***
Torrance	1.062	.06	17.67	0	.944	1.18	***
Union	.792	.118	6.71	0	.561	1.023	***
Valencia	.816	.031	26.10	0	.755	.878	***
Constant	-.274	.052	-5.22	0	-.376	-.171	***

Mean dependent var	0.393	SD dependent var	0.488
Pseudo r-squared	0.152	Number of obs	269369
Chi-square	39277.859	Prob > chi2	0.000
Akaike crit. (AIC)	306297.593	Bayesian crit. (BIC)	307137.900

**** p<.01, ** p<.05, * p<.1*
