# Supplementary material for: Should I vote-by-mail or in person? The impact of COVID-19 risk factors and partisanship on vote mode decisions in the 2020 presidential election
Source: PLoS One. 2022 Sep 15;17(9):e0274357. doi: 10.1371/journal.pone.0274357 (PMC9477279; doi:10.1371/journal.pone.0274357)
Supplement: S13 Table — (PDF) [file pone.0274357.s013.pdf]

S13 Table. Multinomial Logistic Regression Vote Mode Primary Election 2016 (Fig 5g)

	Coef.	SE	t-value	p-value	[95% Conf	Interval]	Sig
VBM							
Age Categories							
30-39 y/o	.614	.1	-3.00	.003	.446	.844	***
40-49 y/o	.55	.085	-3.88	0	.407	.744	***
50-64 y/o	1.001	.128	0.01	.993	.779	1.287	
65-74 y/o	2.005	.255	5.46	0	1.562	2.574	***
75-84 y/o	3.389	.441	9.37	0	2.625	4.374	***
85+ y/o	7.608	1.086	14.21	0	5.751	10.064	***
Political Party							
Democrat	1.643	.395	2.07	.039	1.026	2.631	**
Age X Party							
30-39 X Dem	.901	.293	-0.32	.748	.477	1.703	
40-49 X Dem	.974	.282	-0.09	.928	.552	1.72	
50-64 X Dem	.923	.228	-0.33	.745	.569	1.496	
65-74 X Dem	.843	.208	-0.69	.489	.521	1.366	
75-84 X Dem	.727	.181	-1.28	.201	.446	1.185	
85+ X Dem	.703	.188	-1.32	.187	.416	1.187	
Hispanic	1.042	.037	1.16	.245	.972	1.116	
Asian	1.099	.258	0.40	.688	.694	1.74	
Black	.925	.165	-0.44	.661	.652	1.312	
Other Race	.621	.074	-4.02	0	.492	.783	***
Female	1.316	.041	8.78	0	1.238	1.4	***
Other Sex	0	0	-0.00	.999	0	.	
County							
Catron	.231	.062	-5.42	0	.136	.392	***
Chaves	.153	.022	-13.28	0	.116	.202	***
Cibola	.302	.049	-7.45	0	.22	.413	***
Colfax	.046	.017	-8.09	0	.022	.096	***
Curry	.029	.012	-8.62	0	.013	.065	***
De Baca	.152	.078	-3.66	0	.055	.417	***
Dona Ana	.19	.017	-18.51	0	.159	.226	***
Eddy	0	0	-0.03	.978	0	.	
Grant	.38	.04	-9.26	0	.31	.467	***
Guadalupe	.776	.139	-1.42	.156	.546	1.102	
Harding	.488	.176	-1.99	.047	.24	.991	**
Hidalgo	0	0	-0.01	.992	0	.	
Lea	.191	.028	-11.10	0	.142	.256	***
Lincoln	.21	.037	-8.91	0	.149	.296	***
Los Alamos	.231	.044	-7.69	0	.159	.336	***
Luna	.212	.043	-7.71	0	.143	.315	***
McKinley	.097	.018	-12.65	0	.067	.139	***
Mora	.514	.083	-4.13	0	.375	.705	***
Otero	.219	.027	-12.34	0	.172	.279	***
Quay	3.003	.291	11.33	0	2.482	3.632	***
Rio Arriba	.196	.022	-14.27	0	.156	.245	***
Roosevelt	.737	.087	-2.60	.009	.586	.928	***
San Juan	.517	.034	-9.97	0	.455	.589	***
San Miguel	.275	.032	-10.95	0	.218	.346	***
Sandoval	.653	.039	-7.09	0	.581	.735	***
Santa Fe	.252	.015	-22.49	0	.223	.284	***
Sierra	.25	.051	-6.84	0	.168	.372	***
Socorro	.461	.062	-5.75	0	.354	.6	***
Taos	.017	.007	-9.91	0	.008	.038	***

Torrance	.36	.055	-6.66	0	.267	.487	***
Union	.411	.118	-3.11	.002	.235	.721	***
Valencia	.368	.032	-11.52	0	.311	.437	***
Constant	.113	.014	-17.31	0	.088	.144	***
Early Vote							
Age Categories							
30-39 y/o	.806	.052	-3.36	.001	.711	.914	***
40-49 y/o	.774	.047	-4.22	0	.688	.872	***
50-64 y/o	1.115	.06	2.02	.043	1.003	1.239	**
65-74 y/o	1.785	.097	10.70	0	1.605	1.985	***
75-84 y/o	1.908	.109	11.32	0	1.706	2.133	***
85+ y/o	1.847	.138	8.23	0	1.596	2.137	***
Political Party							
Democrat	.607	.079	-3.84	0	.471	.783	***
Age X Party							
30-39 X Dem	1.166	.185	0.97	.334	.854	1.592	
40-49 X Dem	1.398	.202	2.31	.021	1.052	1.856	**
50-64 X Dem	1.381	.183	2.44	.015	1.066	1.789	**
65-74 X Dem	1.46	.194	2.85	.004	1.126	1.894	***
75-84 X Dem	1.403	.19	2.49	.013	1.075	1.83	**
85+ X Dem	1.276	.203	1.54	.125	.935	1.743	
Hispanic	.728	.012	-19.01	0	.704	.752	***
Asian	1.077	.116	0.69	.49	.872	1.33	
Black	.892	.073	-1.39	.166	.759	1.048	
Other Race	.729	.034	-6.71	0	.665	.8	***
Female	1.051	.015	3.44	.001	1.022	1.082	***
Other Sex	.802	.744	-0.24	.812	.13	4.943	
County							
Catron	.312	.039	-9.42	0	.245	.398	***
Chaves	.482	.023	-15.22	0	.438	.529	***
Cibola	.352	.025	-14.46	0	.306	.405	***
Colfax	.201	.018	-18.02	0	.169	.24	***
Curry	.356	.024	-15.37	0	.312	.406	***
De Baca	.172	.04	-7.63	0	.109	.27	***
Dona Ana	.624	.019	-15.47	0	.588	.663	***
Eddy	.448	.024	-14.96	0	.403	.498	***
Grant	.55	.026	-12.69	0	.501	.603	***
Guadalupe	.273	.036	-9.80	0	.211	.354	***
Harding	.327	.073	-4.99	0	.211	.507	***
Hidalgo	.395	.059	-6.19	0	.295	.53	***
Lea	.396	.023	-15.90	0	.353	.443	***
Lincoln	.353	.026	-14.23	0	.306	.408	***
Los Alamos	.879	.052	-2.18	.029	.783	.987	**
Luna	.602	.043	-7.06	0	.523	.693	***
McKinley	.259	.013	-26.42	0	.234	.286	***
Mora	.439	.038	-9.45	0	.37	.521	***
Otero	.495	.024	-14.66	0	.451	.544	***
Quay	0	0	-0.00	1	0	.	
Rio Arriba	.334	.015	-25.15	0	.307	.364	***
Roosevelt	.05	.009	-16.71	0	.035	.071	***
San Juan	.363	.014	-26.35	0	.337	.392	***
San Miguel	.321	.017	-21.72	0	.29	.356	***
Sandoval	.881	.026	-4.21	0	.831	.935	***
Santa Fe	.526	.013	-26.87	0	.502	.551	***
Sierra	.46	.039	-9.24	0	.39	.542	***
Socorro	.435	.031	-11.75	0	.379	.5	***

Taos	.475	.02	-17.38	0	.437	.517	***
Torrance	.33	.027	-13.67	0	.282	.387	***
Union	.489	.069	-5.04	0	.371	.646	***
Valencia	.392	.017	-21.63	0	.36	.427	***
Constant	1.195	.064	3.32	.001	1.075	1.327	***

Mean dependent var	2.452	SD dependent var	0.602
Pseudo r-squared	0.071	Number of obs	89790
Chi-square	11128.609	Prob > chi2	0.000
Akaike crit. (AIC)	145292.113	Bayesian crit. (BIC)	146270.257

*** *p<.01,* ** *p<.05,* * *p<.1*
