# Supplementary material for: Should I vote-by-mail or in person? The impact of COVID-19 risk factors and partisanship on vote mode decisions in the 2020 presidential election
Source: PLoS One. 2022 Sep 15;17(9):e0274357. doi: 10.1371/journal.pone.0274357 (PMC9477279; doi:10.1371/journal.pone.0274357)
Supplement: S14 Table — (PDF) [file pone.0274357.s014.pdf]

S14 Table. Multinomial Logistic Regression Vote Mode Primary Election 2018 (Fig 5h)

	Coef.	SE	t-value	p-value	[95% Conf	Interval]	Sig
VBM							
Age Categories							
30-39 y/o	.699	.118	-2.12	.034	.503	.973	**
40-49 y/o	.599	.098	-3.13	.002	.434	.826	***
50-64 y/o	1.154	.155	1.07	.287	.887	1.501	
65-74 y/o	2.39	.318	6.54	0	1.841	3.102	***
75-84 y/o	4.299	.583	10.75	0	3.295	5.609	***
85+ y/o	10.885	1.596	16.29	0	8.167	14.508	***
Political Party							
Democrat	2.243	.575	3.15	.002	1.356	3.708	***
Age X Party							
30-39 X Dem	.655	.234	-1.18	.237	.325	1.32	
40-49 X Dem	.785	.247	-0.77	.442	.424	1.454	
50-64 X Dem	.69	.182	-1.41	.159	.411	1.157	
65-74 X Dem	.624	.164	-1.80	.072	.373	1.044	*
75-84 X Dem	.47	.125	-2.84	.004	.279	.791	***
85+ X Dem	.536	.151	-2.21	.027	.309	.931	**
Hispanic	1.054	.04	1.40	.162	.979	1.135	
Asian	.947	.241	-0.21	.83	.574	1.561	
Black	.996	.182	-0.02	.983	.696	1.425	
Other Race	.63	.08	-3.63	0	.491	.808	***
Female	1.321	.044	8.36	0	1.238	1.411	***
Other Sex	0	.027	-0.01	.989	0	.	
County							
Catron	.304	.074	-4.87	0	.189	.491	***
Chaves	.202	.027	-11.87	0	.155	.263	***
Cibola	.204	.04	-8.09	0	.139	.3	***
Colfax	.079	.022	-8.96	0	.045	.138	***
Curry	.022	.011	-7.54	0	.008	.06	***
De Baca	.156	.08	-3.61	0	.057	.427	***
Dona Ana	.121	.013	-19.86	0	.098	.149	***
Eddy	.023	.009	-9.88	0	.011	.049	***
Grant	.395	.042	-8.76	0	.321	.487	***
Guadalupe	1.362	.234	1.80	.072	.973	1.907	*
Harding	.798	.26	-0.69	.489	.421	1.512	
Hidalgo	.293	.109	-3.31	.001	.142	.606	***
Lea	.251	.034	-10.29	0	.193	.327	***
Lincoln	.184	.033	-9.41	0	.13	.262	***
Los Alamos	.208	.042	-7.85	0	.14	.308	***
Luna	.18	.038	-8.06	0	.119	.273	***
McKinley	.052	.013	-11.66	0	.031	.085	***
Mora	.816	.122	-1.36	.173	.609	1.093	
Otero	.257	.031	-11.25	0	.203	.326	***
Quay	.105	.038	-6.23	0	.052	.214	***
Rio Arriba	.152	.021	-13.62	0	.116	.199	***
Roosevelt	.076	.027	-7.18	0	.037	.153	***
San Juan	.551	.038	-8.67	0	.481	.63	***
San Miguel	.355	.039	-9.42	0	.286	.44	***
Sandoval	.499	.032	-10.70	0	.44	.567	***
Santa Fe	.191	.013	-24.30	0	.167	.218	***
Sierra	.284	.06	-5.96	0	.187	.429	***
Socorro	.299	.049	-7.38	0	.217	.412	***
Taos	.075	.015	-12.70	0	.05	.112	***

Torrance	.326	.054	-6.82	0	.236	.45	***
Union	.567	.146	-2.20	.028	.342	.941	**
Valencia	.353	.032	-11.36	0	.295	.422	***
Constant	.089	.012	-18.41	0	.068	.115	***
Early Vote							
Age Categories							
30-39 y/o	.951	.063	-0.76	.449	.836	1.082	
40-49 y/o	1.118	.069	1.80	.071	.99	1.262	*
50-64 y/o	1.692	.094	9.51	0	1.518	1.886	***
65-74 y/o	2.694	.15	17.82	0	2.416	3.004	***
75-84 y/o	2.799	.164	17.62	0	2.496	3.139	***
85+ y/o	2.487	.188	12.04	0	2.144	2.885	***
Political Party							
Democrat	1.349	.163	2.48	.013	1.064	1.709	**
Age X Party							
30-39 X Dem	.823	.122	-1.32	.188	.615	1.1	
40-49 X Dem	.736	.1	-2.27	.023	.564	.959	**
50-64 X Dem	.624	.077	-3.84	0	.49	.794	***
65-74 X Dem	.623	.077	-3.83	0	.489	.794	***
75-84 X Dem	.605	.077	-3.97	0	.472	.775	***
85+ X Dem	.668	.101	-2.66	.008	.496	.9	***
Hispanic	.85	.014	-9.78	0	.823	.878	***
Asian	.979	.105	-0.20	.842	.794	1.208	
Black	.936	.077	-0.81	.418	.797	1.099	
Other Race	.75	.035	-6.18	0	.685	.822	***
Female	1.007	.015	0.46	.644	.979	1.036	
Other Sex	6.887	7.641	1.74	.082	.783	60.592	*
County							
Catron	.291	.036	-9.85	0	.228	.372	***
Chaves	.607	.029	-10.58	0	.553	.666	***
Cibola	.346	.025	-14.95	0	.301	.397	***
Colfax	.115	.012	-20.92	0	.094	.141	***
Curry	.471	.03	-11.66	0	.415	.535	***
De Baca	.159	.037	-7.86	0	.1	.251	***
Dona Ana	.492	.015	-23.25	0	.463	.522	***
Eddy	.333	.018	-19.84	0	.299	.372	***
Grant	.551	.026	-12.69	0	.503	.604	***
Guadalupe	.551	.065	-5.06	0	.438	.694	***
Harding	.437	.093	-3.88	0	.288	.664	***
Hidalgo	.444	.066	-5.46	0	.332	.594	***
Lea	.365	.021	-17.17	0	.325	.409	***
Lincoln	.243	.019	-18.26	0	.209	.283	***
Los Alamos	.83	.049	-3.15	.002	.74	.932	***
Luna	.503	.036	-9.55	0	.437	.579	***
McKinley	.258	.013	-26.71	0	.233	.285	***
Mora	.578	.049	-6.47	0	.49	.682	***
Otero	.556	.026	-12.37	0	.507	.61	***
Quay	.505	.047	-7.33	0	.421	.606	***
Rio Arriba	.433	.018	-20.28	0	.4	.47	***
Roosevelt	.368	.032	-11.33	0	.31	.438	***
San Juan	.46	.017	-20.88	0	.427	.494	***
San Miguel	.312	.016	-22.50	0	.282	.345	***
Sandoval	.712	.021	-11.46	0	.671	.754	***
Santa Fe	.446	.011	-33.64	0	.426	.468	***
Sierra	.641	.053	-5.38	0	.545	.754	***
Socorro	.404	.028	-12.97	0	.352	.463	***

Taos	.443	.019	-18.99	0	.407	.482	***
Torrance	.335	.027	-13.72	0	.287	.392	***
Union	.48	.068	-5.18	0	.363	.634	***
Valencia	.411	.017	-20.91	0	.378	.447	***
Constant	.822	.045	-3.57	0	.737	.915	***

Mean dependent var	2.459	SD dependent var	0.590
Pseudo r-squared	0.061	Number of obs	89790
Chi-square	9387.291	Prob > chi2	0.000
Akaike crit. (AIC)	144238.814	Bayesian crit. (BIC)	145216.958

*** *p<.01,* ** *p<.05,* * *p<.1*
