# Supplementary material for: Should I vote-by-mail or in person? The impact of COVID-19 risk factors and partisanship on vote mode decisions in the 2020 presidential election
Source: PLoS One. 2022 Sep 15;17(9):e0274357. doi: 10.1371/journal.pone.0274357 (PMC9477279; doi:10.1371/journal.pone.0274357)
Supplement: S15 Table — (PDF) [file pone.0274357.s015.pdf]

S15 Table. Multinomial Logistic Regression Vote Mode Primary Election 2020 (Fig 5i)

	Coef.	SE	t-value	p-value	[95% Conf	Interval]	Sig
VBM							
Age Categories							
30-39 y/o	1.041	.078	0.54	.59	.899	1.205	
40-49 y/o	1.222	.087	2.83	.005	1.064	1.405	***
50-64 y/o	1.957	.125	10.50	0	1.727	2.219	***
65-74 y/o	4.56	.306	22.61	0	3.998	5.201	***
75-84 y/o	4.994	.373	21.56	0	4.315	5.78	***
85+ y/o	7.205	.892	15.95	0	5.653	9.183	***
Political Party							
Democrat	.298	.041	-8.72	0	.227	.391	***
Age X Party							
30-39 X Dem	.65	.111	-2.52	.012	.465	.909	**
40-49 X Dem	.709	.111	-2.20	.027	.523	.963	**
50-64 X Dem	.748	.106	-2.05	.041	.566	.988	**
65-74 X Dem	.65	.094	-2.98	.003	.49	.863	***
75-84 X Dem	1.046	.159	0.29	.769	.777	1.408	
85+ X Dem	1.315	.276	1.31	.191	.872	1.984	
Hispanic	.515	.012	-28.28	0	.492	.539	***
Asian	1.004	.168	0.02	.981	.724	1.393	
Black	1.069	.136	0.52	.601	.833	1.372	
Other Race	.519	.029	-11.62	0	.464	.579	***
Female	1.263	.025	11.62	0	1.214	1.314	***
Other Sex	1.831	1.398	0.79	.428	.41	8.18	
County							
Catron	.201	.029	-11.30	0	.153	.266	***
Chaves	.31	.021	-17.65	0	.273	.354	***
Cibola	.299	.025	-14.26	0	.253	.353	***
Colfax	.337	.032	-11.41	0	.28	.407	***
Curry	.24	.019	-17.59	0	.205	.282	***
De Baca	.194	.042	-7.57	0	.127	.296	***
Dona Ana	.296	.012	-28.97	0	.272	.321	***
Eddy	.278	.019	-18.37	0	.242	.318	***
Grant	.331	.022	-16.97	0	.291	.376	***
Guadalupe	.551	.086	-3.83	0	.406	.748	***
Harding	1.033	.283	0.12	.905	.604	1.769	
Hidalgo	4197965.6	1.902e+09	0.03	.973	0	.	
Lea	.342	.027	-13.69	0	.294	.399	***
Lincoln	.477	.044	-7.97	0	.397	.572	***
Los Alamos	1.179	.128	1.52	.129	.953	1.458	
Luna	.385	.039	-9.43	0	.315	.469	***
McKinley	.197	.011	-28.55	0	.176	.22	***
Mora	.21	.02	-16.11	0	.174	.254	***
Otero	.356	.022	-16.49	0	.315	.402	***
Quay	.252	.028	-12.24	0	.202	.315	***
Rio Arriba	.251	.012	-27.77	0	.227	.276	***
Roosevelt	.209	.023	-14.37	0	.169	.259	***
San Juan	.47	.023	-15.37	0	.427	.518	***
San Miguel	.445	.028	-12.82	0	.394	.504	***
Sandoval	.571	.025	-12.93	0	.524	.621	***
Santa Fe	.673	.026	-10.19	0	.623	.726	***
Sierra	.468	.055	-6.51	0	.373	.589	***
Socorro	.335	.028	-12.88	0	.283	.395	***
Taos	.36	.021	-17.20	0	.32	.404	***

Torrance	.383	.036	-10.12	0	.318	.462	***
Union	.298	.052	-6.96	0	.212	.419	***
Valencia	.609	.036	-8.46	0	.543	.683	***
Constant	5.444	.352	26.17	0	4.795	6.18	***
Early Vote							
Age Categories							
30-39 y/o	.759	.092	-2.28	.022	.599	.962	**
40-49 y/o	1.414	.153	3.21	.001	1.144	1.748	***
50-64 y/o	2.428	.237	9.08	0	2.005	2.941	***
65-74 y/o	4.333	.435	14.61	0	3.56	5.275	***
75-84 y/o	4.138	.447	13.14	0	3.348	5.114	***
85+ y/o	3.983	.654	8.42	0	2.888	5.494	***
Political Party							
Democrat	1.964	.303	4.37	0	1.451	2.658	***
Age X Party							
30-39 X Dem	1.117	.211	0.59	.558	.771	1.619	
40-49 X Dem	.712	.123	-1.97	.049	.508	.998	**
50-64 X Dem	.648	.102	-2.75	.006	.476	.883	***
65-74 X Dem	.536	.086	-3.89	0	.391	.734	***
75-84 X Dem	.639	.108	-2.64	.008	.458	.891	***
85+ X Dem	.535	.131	-2.56	.01	.331	.864	**
Hispanic	.905	.025	-3.58	0	.857	.956	***
Asian	.807	.176	-0.98	.326	.527	1.237	
Black	1.18	.17	1.15	.25	.89	1.565	
Other Race	.751	.052	-4.17	0	.656	.859	***
Female	1.065	.025	2.68	.007	1.017	1.115	***
Other Sex	2.415	2.117	1.01	.315	.433	13.468	
County							
Catron	.426	.065	-5.59	0	.316	.575	***
Chaves	1.203	.076	2.90	.004	1.062	1.362	***
Cibola	.538	.057	-5.87	0	.438	.662	***
Colfax	.165	.028	-10.52	0	.118	.231	***
Curry	.568	.048	-6.76	0	.482	.67	***
De Baca	.283	.075	-4.75	0	.168	.476	***
Dona Ana	.808	.039	-4.45	0	.736	.888	***
Eddy	.782	.056	-3.45	.001	.679	.899	***
Grant	.93	.068	-0.99	.324	.806	1.074	
Guadalupe	1.245	.221	1.23	.217	.879	1.764	
Harding	.444	.165	-2.19	.029	.214	.92	**
Hidalgo	11784094	5.338e+09	0.04	.971	0	.	
Lea	1.205	.09	2.50	.012	1.041	1.394	**
Lincoln	.619	.065	-4.57	0	.504	.76	***
Los Alamos	.968	.128	-0.24	.809	.747	1.255	
Luna	1.056	.114	0.51	.612	.855	1.304	
McKinley	.446	.033	-10.97	0	.386	.515	***
Mora	.556	.065	-5.00	0	.442	.7	***
Otero	.737	.049	-4.57	0	.647	.84	***
Quay	.373	.05	-7.37	0	.287	.485	***
Rio Arriba	.469	.03	-11.65	0	.413	.533	***
Roosevelt	.644	.067	-4.22	0	.525	.79	***
San Juan	.756	.041	-5.12	0	.679	.841	***
San Miguel	.573	.048	-6.59	0	.486	.676	***
Sandoval	.681	.036	-7.25	0	.614	.756	***
Santa Fe	.821	.04	-4.05	0	.746	.903	***
Sierra	.855	.107	-1.25	.21	.669	1.092	
Socorro	.528	.053	-6.32	0	.433	.644	***

Taos	.864	.061	-2.05	.04	.752	.993	**
Torrance	.447	.051	-7.03	0	.357	.56	***
Union	.684	.125	-2.08	.038	.478	.979	**
Valencia	.826	.056	-2.82	.005	.724	.943	***
Constant	.49	.048	-7.25	0	.404	.595	***

Mean dependent var	1.503	SD dependent var	0.757
Pseudo r-squared	0.124	Number of obs	89789
Chi-square	19560.953	Prob > chi2	0.000
Akaike crit. (AIC)	138338.299	Bayesian crit. (BIC)	139316.442

**** p<.01, ** p<.05, * p<.1*
