# Supplementary material for: Should I vote-by-mail or in person? The impact of COVID-19 risk factors and partisanship on vote mode decisions in the 2020 presidential election
Source: PLoS One. 2022 Sep 15;17(9):e0274357. doi: 10.1371/journal.pone.0274357 (PMC9477279; doi:10.1371/journal.pone.0274357)
Supplement: S16 Table — (PDF) [file pone.0274357.s016.pdf]

S16 Table. Hazard Model comparing Likelihood to Vote Early by Day across 2018 and 2020 (Fig 7)

Weibull AFT regression

Days	Coef.	SE	t-value	p-value	[95% Conf	Interval]	Sig
Age Categories							
30-39 y/o	-.024	.006	-3.94	0	-.036	-.012	***
40-49 y/o	-.062	.006	-10.62	0	-.073	-.05	***
50-64 y/o	-.166	.005	-32.68	0	-.176	-.156	***
65-74 y/o	-.328	.005	-62.42	0	-.338	-.317	***
75-84 y/o	-.309	.006	-51.10	0	-.321	-.297	***
85+ y/o	-.267	.009	-27.69	0	-.286	-.248	***
Political Party							
Independent	.136	.01	13.32	0	.116	.156	***
Republican	.017	.007	2.34	.019	.002	.032	*
Age X Party							
30-39 X Ind	-.025	.012	-1.99	.047	-.051	-.001	*
30-39 X Rep	.009	.009	1.01	.313	-.001	.028	
40-49 X Ind	-.071	.012	-5.65	0	-.095	-.046	***
40-49 X Rep	-.017	.008	-1.86	.063	-.034	.001	
50-64 X Ind	-.064	.011	-5.78	0	-.086	-.042	***
50-64 X Rep	-.006	.007	-.8	.425	-.021	.009	
65-74 X Ind	-.033	.012	-2.85	.004	-.056	-.01	**
65-74 X Rep	.041	.008	5.04	0	.025	.058	***
75-84 X Ind	-.075	.014	-5.06	0	-.104	-.046	***
75-84 X Rep	.015	.009	1.68	.092	-.002	.033	
85+ X Ind	-.119	.027	-4.29	0	-.175	-.065	***
85+ X Rep	-.006	.014	-.44	.657	-.035	.022	
Election Year							
2020	-.317	.002	-195.27	0	-.319	-.313	***
Hispanic	.141	.002	76.18	0	.137	.145	***
Asian	-.012	.009	-1.21	.228	-.03	.007	
Black	.02	.008	2.29	.022	.002	.037	*
Other Race	.249	.005	50.97	0	.239	.259	***
Female	-.009	.002	-5.99	0	-.013	-.006	***
Other Sex	-.085	.057	-1.48	.140	-.199	.028	
Constant	3.487	.004	697.92	0	3.477	3.496	***
/ln_p	.769	.001	515.06	0	3.477	3.496	***
p	2.158	.003			2.151	2.164	
1/p	.463	.001			.462	.464	

Mean dependent var	20.518	SD dependent var	7.653
Number of obs	485185	Chi-square	63821.839
Prob > chi2	0.000	Akaike crit. (AIC)	905619.708

**** p<.01, ** p<.05, * p<.1*
