# Supplementary figures and images for: Should I vote-by-mail or in person? The impact of COVID-19 risk factors and partisanship on vote mode decisions in the 2020 presidential election
Source: PLoS One. 2022 Sep 15;17(9):e0274357. doi: 10.1371/journal.pone.0274357 (PMC9477279; doi:10.1371/journal.pone.0274357)

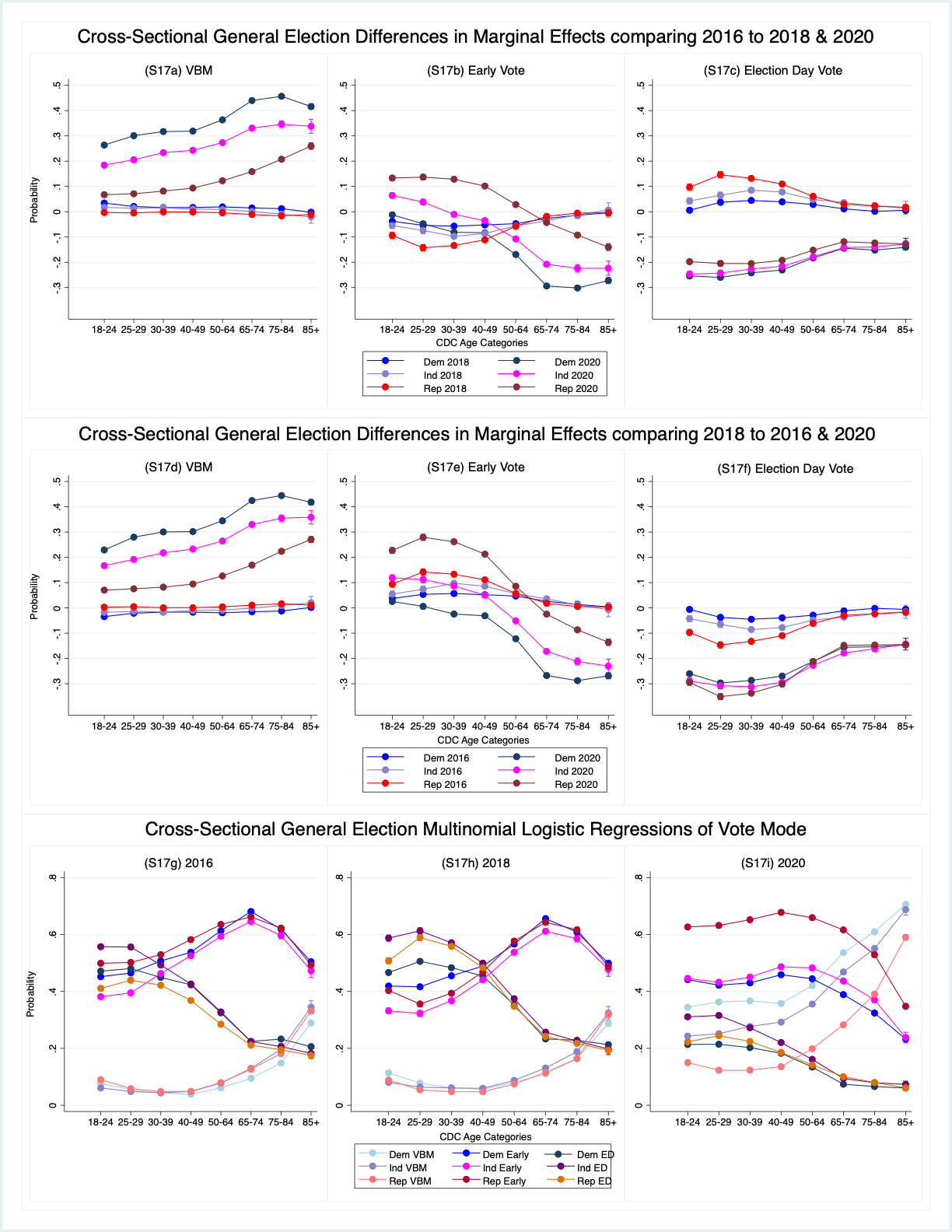

Supplement: S1 Fig — (TIF) [file pone.0274357.s017.tif]

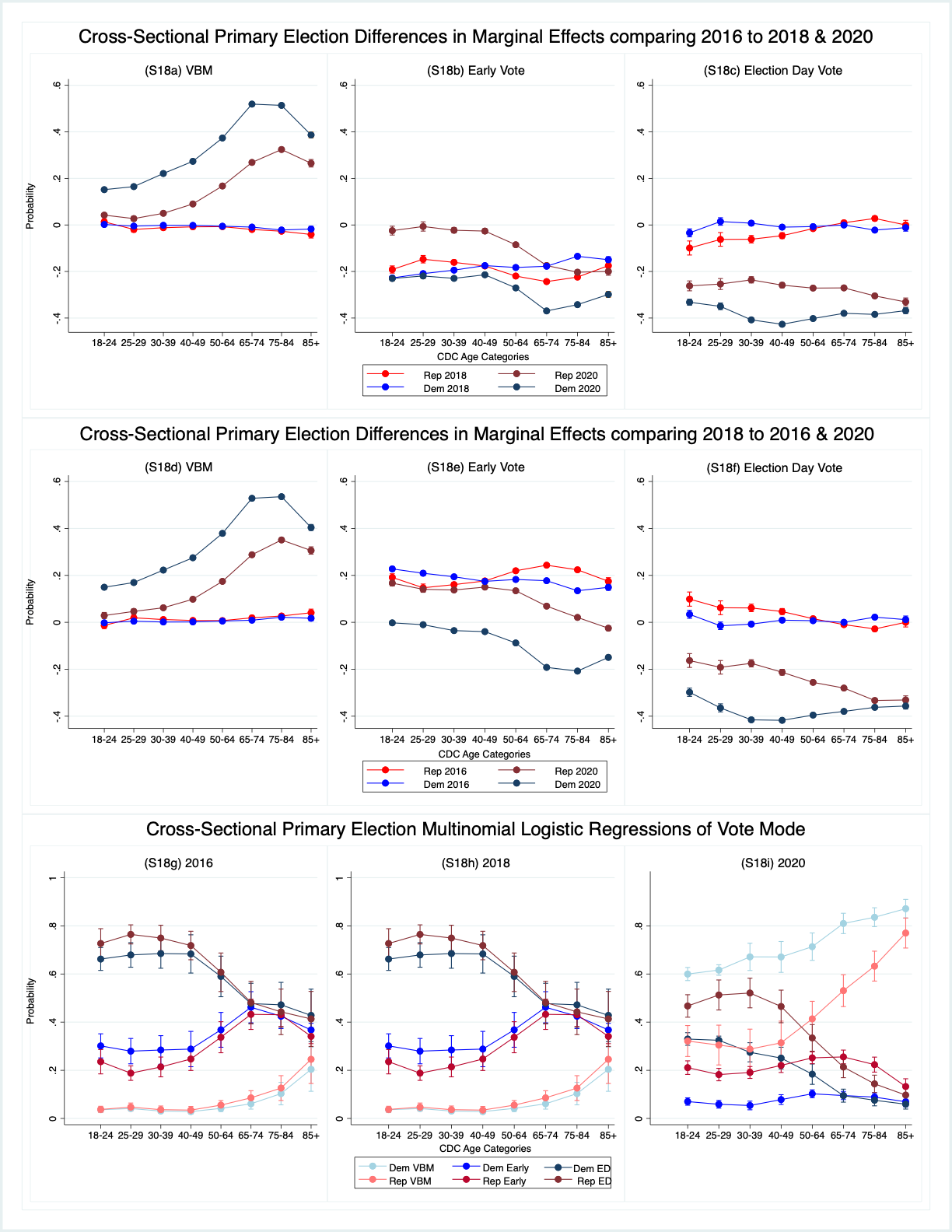

Supplement: S2 Fig — (TIF) [file pone.0274357.s018.tif]
